# Linguistic gender congruity differentially correlates with film and novel ratings by critics and audiences

**DOI:** 10.1371/journal.pone.0248402

**Published:** 2022-04-19

**Authors:** Taleen Nalabandian, Molly E. Ireland

**Affiliations:** Department of Psychological Sciences, Texas Tech University, Lubbock, TX, United States of America; Lancaster University, UNITED KINGDOM

## Abstract

The film and publishing industries are fraught with gender disparities, with men overpowering nearly every sector of these domains. For instance, men are not only paid more than women in the film industry, but they also outnumber women in positions such as director, screenwriter, and lead acting roles. Similarly, women often resort to assuming gender-neutral or male pseudonyms to increase their prospects in the publishing industry. This widespread gender inequality in the film and publishing industries raises the question of how writers’ gender relates to gendered language and narrative receptions. Two archival studies examined whether gender-linked language relates to film (*N* = 521) and novel (*N* = 150) ratings, and whether those associations differ as a function of writer gender or the expertise of the rater (professional critics and lay audience members). Results demonstrated that female screenwriters and novelists used a more feminine style of writing, whereas male screenwriters and novelists used a more masculine style of writing. Lay audiences gave more positive ratings to films and novels by writers who used a more gender-congruent writing style, in contrast with professional critics, who gave more positive reviews to films by writers who used a more gender-incongruent writing style. Our findings substantiate past research regarding the differing tastes of lay audiences and professional critics in addition to lending insight into subtle social dynamics that may sustain gender biases in the film and publishing industries.

## Introduction

Gender issues, including gender biases and inequality, are particularly salient in both the film and publishing industries. Regarding the film industry in particular, allegations of sexual harassment and rape against film producer Harvey Weinstein (currently sentenced to 23 years in prison) kick-started a movement advocating gender equality in the workforce (#timesup; https://www.timesupnow.com), which then helped promote the formerly lesser-known #MeToo movement focusing on sexual violence and harassment more broadly (https://metoomvmt.org). An additional long-standing gender disparity in film includes the unequal pay of actors and actresses. On average, male actors earn a salary of about $11.9 million, whereas female actors earn about $6.6 million, a 45% wage gap [1]. The issue received heavy media coverage after Academy Award-winning actress Jennifer Lawrence wrote and spoke out about her (and other actresses’) salary compared with that of male co-stars (see Letters to Lenny: https://www.lennyletter.com/story/jennifer-lawrence-why-do-i-make-less-than-my-male-costars). Gender inequalities in Hollywood are evident at every level of the film industry’s work force, from crew members to screenwriters and studio heads. Male actors tend to make up the majority of roles in films, while male directors and writers grossly outnumber female directors and writers in production teams [2]. In 2019, women consisted of only 23% of the film staff (e.g., directors, screenwriters, producers, cinematographers) for the top 500 films [3].

Gender disparities are evident not only in the film industry but in publishing as well. The novel has a history of gender disparities comparable to that of film, wherein early novelists were predominantly male, and female writers historically either published anonymously or under a male name. For example, Mary Shelley—despite being the daughter of feminist pioneer Mary Wollstonecraft, author of *A Vindication of the Rights of Woman* (1792), and a staunch advocate for gender equality herself—initially published *Frankenstein* (1818) anonymously. Later, in the early and mid-19^th^ century, female novelists continued the tradition of publishing under male pennames (e.g., Mary Ann Evans as George Eliot, Amantine Lucile Aurore Dupin as George Sand, and the Brontë sisters as Currer, Acton, and Ellis Bell, among others), presumably in order to avoid audience and editorial prejudices [4–6]. At the start of the 20^th^ century in France, Colette greatly increased the reputation of her then-husband as a writer by ghost writing his Claudine stories and novel during the early stages of her career. Even today, women’s literature tends to be marketed very differently than men’s, with feminine colors and iconography (such as women’s clothing or shoes, lipstick, and flowers) featured more prominently on female authors’ covers than the covers of men’s novels over the same topics [7].

Ongoing sexism in the publishing industry has even led some prominent contemporary authors, including perhaps most famously J. K. Rowling, to publish under ambiguous or gender-neutral names. Gender disparities in publishing are likely bidirectionally influenced by readers and publishers, with the public and critical reception of a novel both reflecting and reinforcing publishing biases. For example, marketing female-authored books with feminine covers or advertisements may both cause and be caused by audiences’ expectations of gender congruity in novels. Considering potential mechanisms that contribute to gender inequality within publishing and filmmaking is a small step toward understanding how and why such discrepancies remain prevalent today. Although the present study does not directly examine or manipulate gender inequality in film and publishing industries, we analyze the narrative itself, and determine how gender-relevant features of a narrative relate to its critical and public reception. Such analyses may begin to illuminate the perceptions of narratives that drive decision-making in the industries that produce films and novels.

In particular, fictional narratives offer a glimpse into the gender relations of the real world, serving as a reflection of human behavior, cognition, and emotion. Writers are trained to write about their own perceptions of the world around them; for example, renowned novelist Stephen King encourages budding writers to “write what you know” [8]. Such fictional narratives not only provide insight into the author’s worldview, but also provide insight into the authors themselves as well as their audiences. Past research shows that language use reveals information about an individual’s age, personality, mental health, and gender [9]. In turn, people reading or listening to fictional narratives and linguistic choices made by authors or screenwriters are affected in different ways. For instance, people often accurately predict the characteristics of an author—such as gender—merely by reading a sample of their writing [10]. In addition to human raters accurately identifying author gender through their written work, other research has established similar rates of accuracy using machine learning techniques. An early landmark study [11] demonstrated roughly 80% accuracy in machine learning models classifying author gender based on stylistic features (e.g., function words, parts of speech) in both fictional and non-fictional work. Individuals are also impacted by the fictional universes they are exposed to (in the movies they watch and the books they read), partly through the process of simulating the characters’ fictional experiences [12]. Given that gender disparities dominate nearly every sector of the film and publishing industries, it is important to investigate how the gender of writers relates to how stories are told, and how gender-linked aspects of those narratives are perceived by critics and audiences.

### Gender-linked language

Specifically, the use of computerized text analyses in examining film scripts and novels could potentially reveal linguistic differences in narratives by men versus women, while archival data on critic and audience narrative ratings could elucidate what type of gendered language patterns are favored most. Two common models that attempt to account for gender differences in language use are the biological- and the socialization-based models. The biological model proposes that differences in men’s and women’s behavior can be attributed to biological sex [13]. For example, greater production of testosterone is associated with male-linked behaviors (e.g., dominance, competition, and aggression), while greater production of oxytocin is associated with female-linked behaviors (e.g., pair-bonding and intimacy [14]). Thus, based on the biological perspective, men and women use different language to fulfill divergent goals: Men use language in ways that help them establish dominance and authority, whereas women use language in ways that help them build and sustain relationships [15]. Alternately, socialization-based models (e.g., social role and social-constructionist theories) emphasize the influence of societal strictures and expectations on gender-linked differences in behavior, wherein men’s and women’s behavior corresponds to traditional societal gender roles [14, 15]. In other words, women are more affiliative because their role in society is the nurturing caregiver, and men are more assertive because their role in society is the provider for their family. In fact, gendered language is often categorized in terms of assertiveness or affiliation. Assertive language is defined as words or speech acts that establish a person’s authority and power, and affiliative language fosters positive interactions or relationships [16]. Women tend to use more affiliative language, such as polite (e.g., “please,” “thank you”) and tentative words and phrases (e.g., hedges such as “I guess”), whereas men tend to use more assertive or dominant language (e.g., aggressive, verbal sexual harassment, interruptions).

Scores of studies have established language profiles for men and women in a variety of contexts through different methods of linguistic analysis. Some studies employ coding techniques to differentiate style or function words (grammatical language categories, such as pronouns, articles, and prepositions) from content words (language expressing the topic or tone of conversation, such as positive and negative emotions words) in individuals’ language use [9]. Other studies utilize more quantitative, automated methods, such as dictionary-based computerized text analysis (e.g., Linguistic Inquiry and Word Count; LIWC), which determines the rates of language categories used in a text by comparing words in said text to a large number of internal dictionaries or word lists. Topic modeling methods are another way of analyzing language, and typically involve identifying common topics or themes in a sample of texts based on co-occurrences of words in the texts themselves (e.g., latent Dirichlet allocation; LDA, [17]). Thus, a large body of research applying various methodologies from linguistics, computer science, and psychology suggests that specific language categories are used differently by women and men.

The overall pattern of findings based on such interdisciplinary work intimate that women and men tend to use affiliative versus assertive language, respectively. For example, researchers coded emails and letters in terms of style and content and found that women were more likely to use language surrounding family, shopping, clubs, and positive emotion, whereas men were more likely to use more offensive language [18]. Other researchers [15] used computerized text analyses (e.g., LIWC) to examine transcripts of couples talking about specific marital issues (e.g., infidelity) with their therapist. Results showed that women were more likely to use social words than men, and men were more likely to use self-references than women. Women’s use of social language is consistent with their more affiliative gender role. However, in that study, men’s greater use of self-references is not consistent with their masculine gender role. Previous research has linked high rates of first-person singular pronouns with lower status [19] and greater psychological distress (e.g., depression, neuroticism [20]), both of which are more commonly associated with women. Therefore, it is important to consider the context in which language is spoken or written. Perhaps men’s greater rates of *I*-words reflect lower status, less control, or greater defensiveness, relative to their partners, in therapy sessions focusing on marital conflict (i.e., topics that may trigger negative affect). In other words, because first-person singular pronouns reflect not only gender but also status and negative emotionality, among other psychological dimensions, we should not necessarily expect to see standard gender differences in “I” usage in situations where distress may be more salient than gender.

Similar research utilizing dictionary-based computerized text analyses has examined potentially less conflict-ridden contexts for language, such as personal ads and interviews, finding opposite effects in terms of pronoun use between men and women. For instance, a study examining language used by men and women during interviews from television shows, such as ABC’s *Good Morning America* and NBC’s *Today Show*, found that women used more language focused on social, cognitive (e.g., certainty words), and sensory (e.g., physical words) processes, as well as pronouns (particularly *I*-words); men used more words greater than six letters, articles, and nouns [21]. Another study analyzed personal ads—where people posted a short blurb about themselves and what they were looking for in a mate—and found that women used more first- and third-person singular pronouns, positive emotion, physical, and sexual language, while men used more articles and career-related language [22]. Thus, within a less distressing context, women’s use of first-person singular pronouns and social language in their personal ads and TV interviews may represent affiliative themes, such as interdependent self-construal, or describing themselves in relation to others (e.g., friends, family). In contrast, men’s use of articles and job-related language in the same context represent their less personal and more object-focused language profile, consistent with linguistic findings from research analyzing academic texts [23].

In addition to the closed-vocabulary or dictionary-based methods used by the aforementioned studies, other research on gender and language has employed an open-vocabulary, data-driven approach to language analysis. Using LDA—a topic modeling method which derives topics or concepts based on clusters of words that co-occur in a given text—researchers examined the language used in men’s and women’s Facebook status messages [24]. Results showed that women used more adverbs as well as more language indicative of positive emotion and relationships, whereas men used more language relating to politics, sports, competition, and shared activities. By correlating the Facebook users’ scores on extraversion and agreeableness with gender-linked language categories, the same researchers were able to classify each language category as representative of affiliation or assertiveness and found that female-linked language was more often classified as affiliative than male-linked language. Although there were no differences in gender-linked language and assertiveness, women used warmer language (i.e., positive emotion), while men used more negative or colder language (i.e., swearing, criticism). Another study where language use on Facebook was analyzed using similar open-vocabulary techniques also found that men used more swear words and women used more emotion words as well as more self- and social-references [25]. Together, the literature finds subtle but reliable gender differences in language, with women using more personal and socially sensitive language than men, and men using more impersonal and object-focused or categorical words than women [23].

Furthermore, a large-scale dictionary-based computerized text analysis of 14,000 spoken and written text samples (e.g., stream of consciousness, fiction, college exams, spoken conversations) collected from multiple labs confirmed the aforementioned pattern of gender-linked language found in previous work [26]. Specifically, women used more first- and third-person singular pronouns, negations, verbs, social words (e.g., references to friends and family), psychological process words (e.g., emotions, sensations), cognitive process words (e.g., insight, discrepancy, certainty), and words relating to home. On the other hand, men used more swear words, words relating to sports, numbers, articles, prepositions, words per sentence, and large words (i.e., greater than six letters). A more recent study analyzing nearly 7,000 conversations corroborated past results, finding that women used more first- and third-person singular pronouns, auxiliary verbs, adverbs, and conjunctions, and men used more articles, prepositions, and quantifiers [27]. These linguistic cues of gender coincide with gender stereotypes and previous research elaborating on how language used by women places a greater emphasis on interpersonal connections, whereas language used by men focuses more on objective, assertive statements.

#### Gender-linked language in narrative

A wealth of recent work has focused on computational linguistic approaches to understanding literature, film, and other narrative texts in relation to gender-linked language. Specifically, research evaluating character dialogue of film scripts found that male and female characters use language patterns comparable (but not identical) to those of men and women in real life. For example, in films, male characters use more sophisticated language as well as more words associated with masculinity, achievement, death, and swear words, whereas female characters use more positive emotion words [2]. Notably, language differences between women and men in fiction may be a product of media bias, wherein male and female character roles often embody (or exaggerate) gender stereotypes through their speech and actions [21]. Women in particular are more often than men depicted as embodying a social role, such as love interest or mother, rather than driving the action—a pattern that seems to persist even in roles where women depict scientists or engineers [28–30]. Along the same lines, researchers have studied characters’ dialogue in novels as a way to examine gender-linked differences in language. For instance, the dialogue of male and female characters in Jane Austen’s novel *Pride and Prejudice* was analyzed, demonstrating that female characters on average used more feminine language (e.g., verbs, negations, negative emotion, and certainty words) and male characters used more masculine language (e.g., words greater than six letters, articles, prepositions [31]), consistent with previous literature [26].

Prior research on gender-linked language of fictional characters shows that art seems to imitate or reflect the language of real-life men and women, in many respects. Nevertheless, possible moderating variables, such as writer gender or narrative genre, may play a role in gender-linked language use within fictional narratives. For instance, results indicated that female characters used more feminine language than male characters in dialogue written by both men and women; however, male characters used more masculine language in dialogue written by men rather than by women [27]. In other words, dialogue in scripts appears to illustrate language style congruent with the character’s gender, moderated by the screenwriter’s gender, with more dramatic gender differences seen in scripts written by men. Other research has examined gender ladenness, a measurement of the degree to which language is masculine versus feminine, as a function of gender and film genre [32]. Findings demonstrated that characters in action movies used more masculine language and characters in romance and comedy movies used more feminine language, moderated by screenwriter gender such that female screenwriters used more masculine language in action movies, relative to male screenwriters. Relatedly, other work [11] involving identification of author gender through texts using machine learning demonstrated greater accuracy in models differentiating between genres (fictional versus non-fictional work) rather than genders (female versus male authors). Thus, both writer gender and genre may serve to inform gender-linked language in fiction.

### Hypotheses

The current research explored gender-linked language in film scripts (including both dialogue and screen directions) and novels. More specifically, two archival studies tested whether writer gender coincided with gender-linked language in film scripts and novels. We predicted that—consistent with previous studies on gender differences in language use—female writers would use more feminine language (first- and third-person singular pronouns, adverbs, auxiliary verbs, common verbs, negations, conjunctions, and social words), whereas male writers would use more masculine language (words greater than six letters, quantifiers, numbers, swear words, articles, and prepositions) in their scripts and novels. We chose these language categories as past research—particularly studies analyzing large corpora [26, 27]—has found the largest and most reliable gender effects for those categories.

We also tested whether gender-linked language correlated with film and novel ratings as a function of writer gender or the expertise of the rater (e.g., professional critic, audience member). Past research [2, 27] suggests that gender-congruent language style is typical (at least in dialogue) of film, and typicality in narratives may reflect higher ratings. In other words, what is easier to process is often more enjoyable [33]. Therefore, we predicted that audiences—who drive the film and publishing industries to produce material they will enjoy, ensuring box office and best-seller revenues [34]—would give more positive ratings to narratives that employ language consistent with the gender of the writer. On the other hand, we predicted that critics would prefer narratives that employ language that is *inconsistent* with the gender of the writer, given that violating expectancies may be viewed as complexity or creativity, which professional film critics tend to enjoy [35].

In sum, for both Studies 1 and 2, we predicted that film and novel audiences would prefer narratives written by women incorporating more female-linked (or feminine) language, and narratives written by men incorporating more male-linked (or masculine) language. In Study 1, we predicted that critics would prefer films written by women containing more masculine language and films by men containing more feminine language. Finally, because past research has identified genre as a potential mechanism for gender-linked language in fiction [11, 32], we controlled for narrative genre and topic.

Although we acknowledge that film audiences may be less consciously aware of screenwriter gender than professional film critics, studies have shown individuals’ adeptness for predicting gender and other gender-related characteristics [10, 36]. For audiences, awareness of writer gender is typically more salient in novels (i.e., on the book cover and title page) than in films. Thus, narrative ratings should be more strongly related to gender-linked language and writer gender in Study 2 (novels) than in Study 1 (films). However, the present studies do not experimentally manipulate audience or reader knowledge or awareness of writer gender. Our current findings and interpretations are limited to discussing the naturally occurring patterns among writer gender, rater role, and gender-linked language in fiction.

## Study 1

### Method

In the first study, we conducted archival research using computerized text analysis on a sample of film scripts to determine whether film ratings differed based on the language style and gender of screenwriters as well as the role (audience or critic) of the individual rating the film. Film scripts were obtained from the Internet Movie Script Database, Indie Film Hustle, and Hollywomen, and film ratings were obtained from the Internet Movie Database and Rotten Tomatoes. All data collected are publicly available from the aforementioned websites, and the collection method complied with the terms and conditions for each website. The data collection and sharing procedures of Study 1 are consistent with *PLOS ONE*’s data management and availability policies. The two datasets used to conduct all descriptive and inferential statistical analyses highlighted in Study 1 (*ANOVA Film Data*, for all ANOVA models, and *Linear Mixed-Effects Film Data*, for all linear mixed-effects models) are publicly available on the Open Science Framework (OSF; see https://osf.io/jgcnu/).

#### Sample

The current sample of film scripts was based on a sample from a previous study examining genre-typical language in film [37] and was obtained from the drama category of the Internet Movie Script Database (*n* = 509; IMSDb; https://www.imsdb.com). However, in order to acquire at least 50 film scripts written by women, we gathered additional scripts (*n* = 5; *Young Adult*; *Lady Bird*; *Middle of Nowhere*; *Somewhere*; *The Invisible Woman*) that were not available on IMSDb from alternative script repositories (Indie Film Hustle and Hollywomen). We also updated our previous IMSDb drama script sample with additional drama scripts (*n* = 4; *Belle*, *Room*, *My Girl*, *1492*: *Conquest of Paradise*) as well as scripts from different categories on IMSDb (*n* = 3), such as comedy (*Bridesmaid*; *It’s Complicated*) and horror (*Jennifer’s Body*). Thus, our total sample consisted of 521 film scripts.

We examined scripts mostly from the drama category of IMSDb, because drama appeared to be a sub-genre for most films in the IMSDb drama category (i.e., films in the drama category were also found in other categories on the site). Furthermore, because popular movie websites, like Rotten Tomatoes and the Internet Movie Database (IMDb), label the majority of films acquired from IMSDb’s drama category with multiple genres (e.g., *Blade Runner* as science-fiction/fantasy, thriller, action, and drama), we identified a subgenre (other than drama) that best represented each film in our sample. Two research assistants coded a single sub-genre for each film, and discrepancies were resolved by a third rater. The sample of films was divided into eight sub-genres: action-adventure (*n* = 77), comedy (*n* = 77), family/kids (*n* = 12), history/war (*n* = 70), romance (*n* = 73), science-fiction/fantasy (*n* = 59), thriller/suspense (*n* = 135), and tragedy (*n* = 18). Genre was added as a categorical covariate to our models as a way to determine whether differences in gender-linked language use—as well as differences in film ratings as a function of screenwriter gender, gender-linked language, and rater role—would remain above and beyond film genre.

The films’ year of release ranged from 1932 to 2017 with 85.4% of scripts written by men, 9.6% written by women, and 5% written by male and female scriptwriting teams. Although the percentage of film scripts by women is quite low, the proportion is similar to that of the real-life prevalence of female scriptwriters (i.e., only 14–20% were women writers for the top 500 films spanning from 2016–19 [3, 38–40]). Films were excluded from data collection if they were not in English, included fewer than 1,000 words, or had fewer than 20 ratings from film critics or audiences.

Additionally, we analyzed the language of each script in its entirety, including both dialogue and screen directions. Although some linguistic analyses of film and novels often focus on character dialogue [2, 27, 31, 32], we approached the present analysis more holistically. The aim of our current research was to examine gender-linked differences in narrative and how such differences relate to audience and critic reviews. To capture a complete picture of the linguistic profile of male and female screenwriters, it is essential to examine the language used to introduce the scenes and describe nonverbal actions in addition to character dialogue. Screen directions are not presented to audiences as explicitly as character dialogue. However, screen directions are made apparent through other modes (e.g., actions of the characters, cinematography) and help shape the audience’s response to the narrative. Thus, for the purposes of the present study, we analyzed the full script of each film in our sample.

#### Measures

*Linguistic inquiry and word count*. The Linguistic Inquiry and Word Count (LIWC [41]) software was used to determine the percentages of different language categories for each of the 521 film scripts. LIWC can identify nearly 6,400 words (or word stems, emoticons, etc.) from over 90 language categories. These language categories range from conversational topics (e.g., work, home) and psychological processes (e.g., anxiety, tentativeness) to grammar or function words (e.g., articles, prepositions). However, the present study focused on the language categories that make up feminine (first- and third-person singular pronouns, adverbs, auxiliary verbs, common verbs, negations, conjunctions, and social words) and masculine (words greater than six letters, quantifiers, numbers, swear words, articles, and prepositions) language, as identified in previous research outlined in our Introduction. All gender-linked language categories were normally distributed, except first-person singular pronouns, numbers, and swear words, which were positively skewed. We used the square root transformation for first-person singular pronouns, the log base 10 transformation for numbers, and the reciprocal cube transformation for swear words in order to achieve normality.

Using the female- (feminine) and male- (masculine) linked language categories (see Table 1 for examples of words in each language category), we computed a gender-linked language composite by standardizing (z-scoring) each language category, adding the female-linked categories, subtracting the male-linked categories, and dividing the total frequency by the number of categories for each film script (Cronbach’s α = .28):

$$z_{Gender‐linked\;language\;composite}=(z_{I}+z_{shehe}+z_{auxverb}+z_{adverb}+z_{verb}+z_{conj}+z_{negate}+z_{social}-z_{prep}-z_{article}-z_{quant}-z_{number}-z_{sixltr}-z_{swear})/14$$ (1)

**Table 1:** Examples of LIWC 2015 language categories for statistical analysis.

Language Categories	Examples
**Feminine Language Style**	
1^st^ Person Sing. Pron.	*I*, *me*, *my*
3^rd^ Person Sing. Pron.	*she*, *he*, *him*
Common Verbs	*thrown*, *unfriended*, *questioned*
Adverbs	*already*, *while*, *briefly*
Auxiliary Verbs	*must*, *have*, *become*
Conjunctions	*also*, *because*, *nevertheless*
Negations	*can’t*, *no*, *never*
Social	*sugardaddy*, *party*, *together*
**Masculine Language Style**	
Numbers	*zillion*, *twice*, *quarter*
Words > Six Letters	*language metric*
Quantifiers	*nada*, *bunch*, *less*
Articles	*a*, *an*, *the*
Prepositions	*in*, *about*, *upon*
Swear	*effin*, *arse*, *biatch*
**Personal Concerns**	
Work	*school*, *overtime*, *executive*
Leisure	*marijuana*, *Netflix*, *vacation*
Home	*landlord*, *domestic*, *doghouse*
Money	*shopaholic*, *embezzle*, *lottery*
Religion	*immortal*, *rite*, *demon*
Death	*murder*, *zombie*, *genocide*

Positive scores on the gender-linked language composite suggest higher rates of female-linked (and lower rates of male-linked) language use, whereas negative scores reflect the opposite: higher rates of male-linked (and lower rates of female-linked) language use.

In addition to the female-linked and male-linked language categories, we analyzed topical language categories (work, leisure, home, money, religion, and death; refer to Table 1 for examples). The topical language categories served as continuous covariates for our main effect models examining gender-linked language as a function of screenwriter gender (as well as our interaction effect models examining film ratings as a function of gender-linked language, screenwriter gender, and rater role) so as to establish whether our hypothesized effects held regardless of differing topics exhibited within the film scripts. The work, leisure, money, religion, and death topical language categories were all positively skewed and subsequently transformed (and standardized) using the log base 10 transformation to reach normality. The home language category was also positively skewed, but only necessitated a square root transformation to achieve normality.

*Film ratings*. We collected both audience and professional critic ratings for each film from Rotten Tomatoes (https://www.rottentomatoes.com) and the Internet Movie Database (IMDb; https://www.imdb.com). We chose these sites because they are among the most popular film review websites [42] and both are explicit about the way in which they calculate ratings. In the current study, audience and critic ratings represent a composite score of multiple ratings for each film. Audience ratings consist of user ratings from both Rotten Tomatoes and IMDb. Specifically, Rotten Tomatoes displays the percentage of positive ratings and ratings on a scale of one to five, whereas IMDb includes a weighted average for audience ratings. Each of these three forms of audience ratings were standardized and subsequently averaged into a composite variable. Professional critic ratings were solely obtained from Rotten Tomatoes (percentage of positive ratings and ratings on a scale of one to ten) and were also standardized and averaged into a separate composite variable.

#### Statistical analyses

*Main effects*. In order to assess the main effects of screenwriter gender on language use in film scripts, we conducted a one-way ANOVA in R [43] with screenwriter gender as the explanatory variable and the gender-linked language composite as the outcome variable. Follow-up Tukey HSD tests were also conducted to determine significant linguistic differences between male, female, and mixed-gender screenwriting teams. However, the gender-linked language composite is made up of 14 different language categories, with each language category representing a different aspect of gender. Although female-linked language is generally affiliative and male-linked language is less personal, there are subtle nuances in the representation of each language category, as outlined in our Introduction. For instance, relative to men, women’s language is more polite or socially sensitive (less swearing, more hedges including “I”, more adverbs like “very” and “so”) and affiliative (more “she” and “he”), and conversational (more conjunctions), whereas men’s language is more focused on objects and their relations (more articles, prepositions, and quantifiers). Therefore, as a way to further uncover if there were any particular language categories driving the gender effect, we conducted multiple one-way ANOVAs with follow-up Tukey HSD tests, one ANOVA for each of the gendered language categories as a function of screenwriter gender. We also ran the main effect models as ANCOVAs with the addition of genre as a categorical covariate and the six topical language categories (home, leisure, work, money, death, and religion) as continuous covariates, in order to establish whether the main effects remained after controlling for film genre and topic: *Language Category ~ Screenwriter Gender + Genre + Work + Home + Death + Religion + Leisure + Money*. All models were considered significant if they indicated *p* < .05.

Additionally, because conducting several ANOVAs inflates Type 1 Error rates, we conducted a single one-way MANOVA, testing the main effect of screenwriter gender on the linear combination of all outcome variables (i.e., gender-linked language categories), to help control for inflation. The MANOVA was statistically significant (*p* < .001) and follow-up discriminant analyses demonstrated that the strongest linguistic predictors of screenwriter gender were identical to those illustrating significant effects in the ANOVA analyses. Because the results of the MANOVA revealed comparable results to that of the individual ANOVAs, we reported the main effects of each ANOVA rather than the MANOVA.

*Interaction effects*. To examine film ratings as a function of gendered language use, screenwriter gender, and rater role (audience or critic), we conducted several linear mixed-effects models using the nlme package [44] in R. Specifically, we regressed film ratings on screenwriter gender and rater role for the gender-linked language composite as well as each of the feminine and masculine language categories (e.g., Film Ratings ~ Language Category * Screenwriter Gender * Rater Role). *Film Ratings* was treated as the continuous outcome variable, *Language Category* as the continuous explanatory variable, and *Screenwriter Gender* and *Rater Role* as the two categorical explanatory variables. Follow-up simple slopes tests were conducted using regression models to deconstruct any significant linear mixed-effects interaction models.

As previously stated with regard to the main effect analyses, because each language category represents an independent construct of gender, running separate tests for each category would help uncover whether particular categories are driving any significant effects found within the gender-linked language composite model. We also added genre and the six topical language categories (home, leisure, work, money, death, and religion) as continuous covariates to determine if the interaction effects remained significant regardless of film genre and topic (e.g., Film Ratings ~ Language Category * Screenwriter Gender * Rater Role + Genre + Work + Home + Death + Religion + Leisure + Money).

We did not conduct a three-way MANOVA examining the effect of screenwriter gender, rater role, and gender-linked language on film ratings, because MANOVAs are best used for models with one (or more) categorical independent variable and two (or more) continuous dependent variables [45]. Therefore, because our interaction effect models include both categorical (screenwriter gender and rater role) and continuous (language categories) independent variables, as well as only one dependent variable (film ratings) nested within rater role, we conducted linear mixed-effects models.

### Results

#### Gender differences in film scripts

Partly inconsistent with our hypotheses and previous research concerning linguistic differences between men and women, the one-way ANOVA testing the effect of screenwriter gender on the gender-linked language composite was not statistically significant, *F*(2) = 2.77, *p* = .064, *η*_p_^2^ = .01. However, the follow-up Tukey HSD tests showed a modest trend in the direction of our predictions and past literature, in that female screenwriters scored slightly higher on the gender-linked language composite—using more female-linked and fewer male-linked language categories—than did male screenwriters (*p* = .056, 95% CI [-0.01, 0.69]). Furthermore, to determine whether certain gender-linked language categories may individually contribute a larger effect, we ran separate models for each gender-linked language category in the composite.

Largely coinciding with our hypotheses and past research on gender differences in language, one-way ANOVAs demonstrated significant effects of screenwriter gender on the following feminine language categories: third-person singular pronouns (*F*[2] = 4.39, *p* = .013, *η*_p_^2^ = .02) and social words (*F*[2] = 5.82, *p* = .003, *η*_p_^2^ = .02). Follow-up Tukey tests revealed that female screenwriters used significantly more third-person singular pronouns (*p* = .019, 95% CI [0.05, 0.75]) and social words (*p* = .002, 95% CI [0.15, 0.85]) in their scripts than did male screenwriters. Similarly, one-way ANOVAs exhibited significant effects of screenwriter gender on the following masculine language categories: articles (*F*[2] = 10.50, *p* < .001, *η*_p_^2^ = .04) and prepositions (*F*[2] = 3.22, *p* = .041, *η*_p_^2^ = .01). Tukey tests substantiated that male screenwriters used significantly more articles (*p* < .001, 95% CI [-1.00, -0.31]) and prepositions (*p* = .031, 95% CI [-0.73, -0.03]) in their film scripts than did female screenwriters.

There was a significant effect of screenwriter gender on use of quantifiers in scripts (*F*[2] = 3.39, *p* = .034), but follow-up Tukey tests were not significant (*p*s > .05). However, there was a modest, nonsignificant difference between male screenwriters and mixed-gender screenwriting teams, such that male screenwriters used more quantifiers in their scripts than mixed-gender screenwriting teams (*p* = .084, 95% CI [-0.87, 0.04]). The effect of screenwriter gender on swear word usage in scripts showed a similar trend (*F*[2] = 2.66, *p* = .071, *η*_p_^2^ = .01), with Tukey tests revealing that men used slightly more swear words in their scripts than women did (*p* = .085, 95% CI [-0.03, 0.67]). There were no significant differences (all *p*s > .1) between male and female screenwriters’ use of first-person singular pronouns, auxiliary verbs, common verbs, conjunctions, negations, numbers, and words with more than six letters. See Table 2 for specific mean differences in language use of male and female screenwriters as well as mixed gender screenwriting teams.

**Table 2:** Descriptive statistics for language use in films across female, male, and mixed gender writers.

		Female	Male	Mixed Gender
	LIWC label	*M* (*SD*)	*M* (*SD*)	*M* (*SD*)
*Gender-Linked Language Composite* †		0.30 (0.14)†	-0.04 (0.05)†	0.09 (0.19)
*Feminine Language*				
1^st^-Person Sing. Pron.	i	2.60 (0.13)	2.49 (0.04)	2.55 (0.17)
3^rd^-Person Sing. Pron.*	shehe	4.34* (0.14)	3.93* (0.05)	4.20 (0.19)
Verbs	verb	14.55 (0.27)	14.67 (0.09)	14.85 (0.36)
Adverbs	adverb	3.64 (0.09)	3.63 (0.03)	3.64 (0.12)
Auxiliary verbs	auxverb	6.24 (0.19)	6.33 (0.06)	6.24 (0.25)
Conjunctions	conj	4.15 (0.09)	4.15 (0.03)	3.98 (0.13)
Negations	negate	1.49 (0.06)	1.54 (0.02)	1.56 (0.08)
Social*	social	12.35* (0.21)	11.60* (0.07)	11.76 (0.28)
*Masculine Language*				
Numbers	number	2.08 (0.18)	1.98 (0.06)	2.06 (0.24)
> Six Letters	sixltr	14.70 (0.36)	14.98 (0.12)	15.01 (0.48)
Quantifiers*	quant	1.23 (0.03)	1.28† (0.01)	1.19† (0.04)
Articles*	article	6.95* (0.18)	7.79* (0.06)	7.43 (0.24)
Prepositions*	prep	12.15* (0.17)	12.60* (0.06)	12.57 (0.23)
Swear†	swear	0.24† (0.04)	0.32† (0.01)	0.34 (0.05)

With the exception of the gender-linked language composite, the above means and standard deviations are derived from the non-standardized language categories. All numbers are percentages obtained from LIWC [41].

*p < .05.

†*p* < .1.

#### Professional film critic versus audience preferences

**Fig 1:**
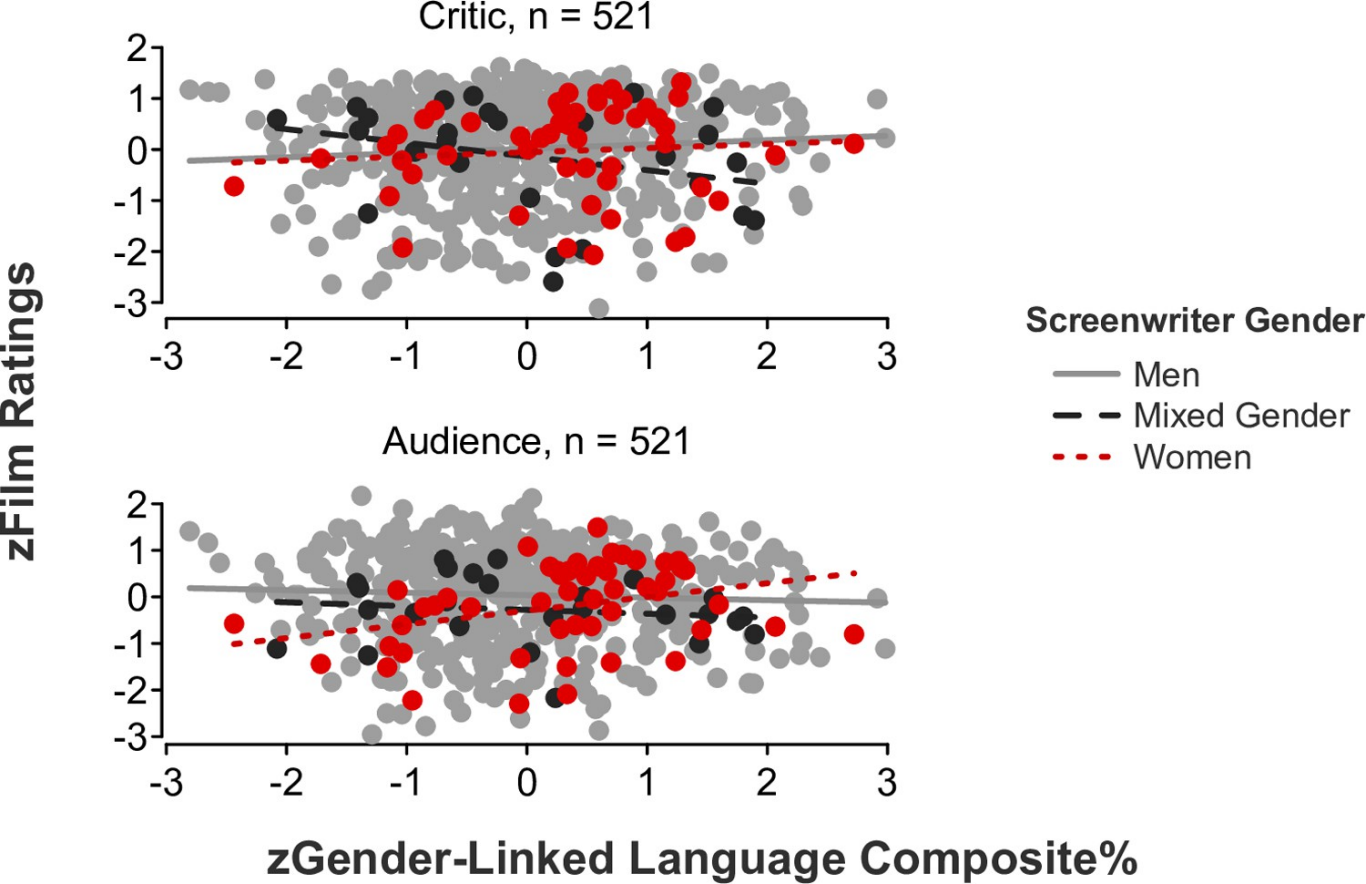
Film ratings as a function of the gender-linked language composite, screenwriter gender, and rater role.

We initially predicted that audiences would give higher ratings to films using language that is more congruent with the screenwriter’s gender, whereas critics would give higher ratings to films using less gender-congruent language. We conducted a linear mixed-effects model regressing film ratings on the gender-linked language composite, screenwriter gender, and rater role, revealing a significant three-way interaction (*b* = -.34, *SE* = .11, *t*[515] = -3.25, *p* = .001, 95% CI [-0.55, -0.13]; Fig 1). Partly consistent with our predictions, follow-up simple slope tests confirmed that audiences gave significantly higher ratings to films written by women who incorporated more feminine and less masculine language (i.e., scoring higher on the gender-linked language composite) in their scripts (*b* = .29, *SE* = .12, *t*[48] = 2.29, *p* = .026, 95% CI [0.04, 0.54]). However, all other simple slopes were nonsignificant (*p*s > .05).

**Fig 2:**
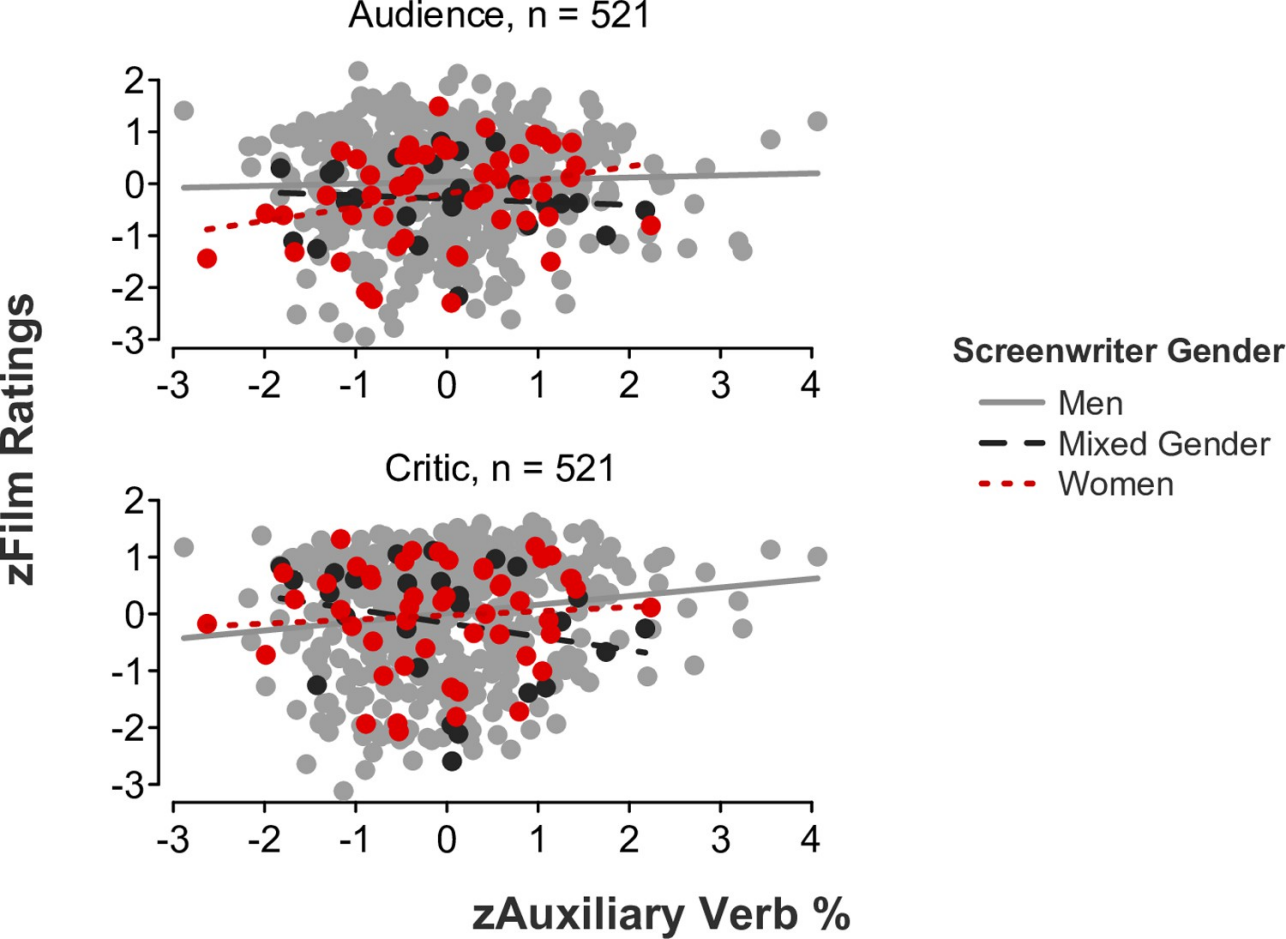
Film ratings as a function of auxiliary verb use, screenwriter gender, and rater role.

**Fig 3:**
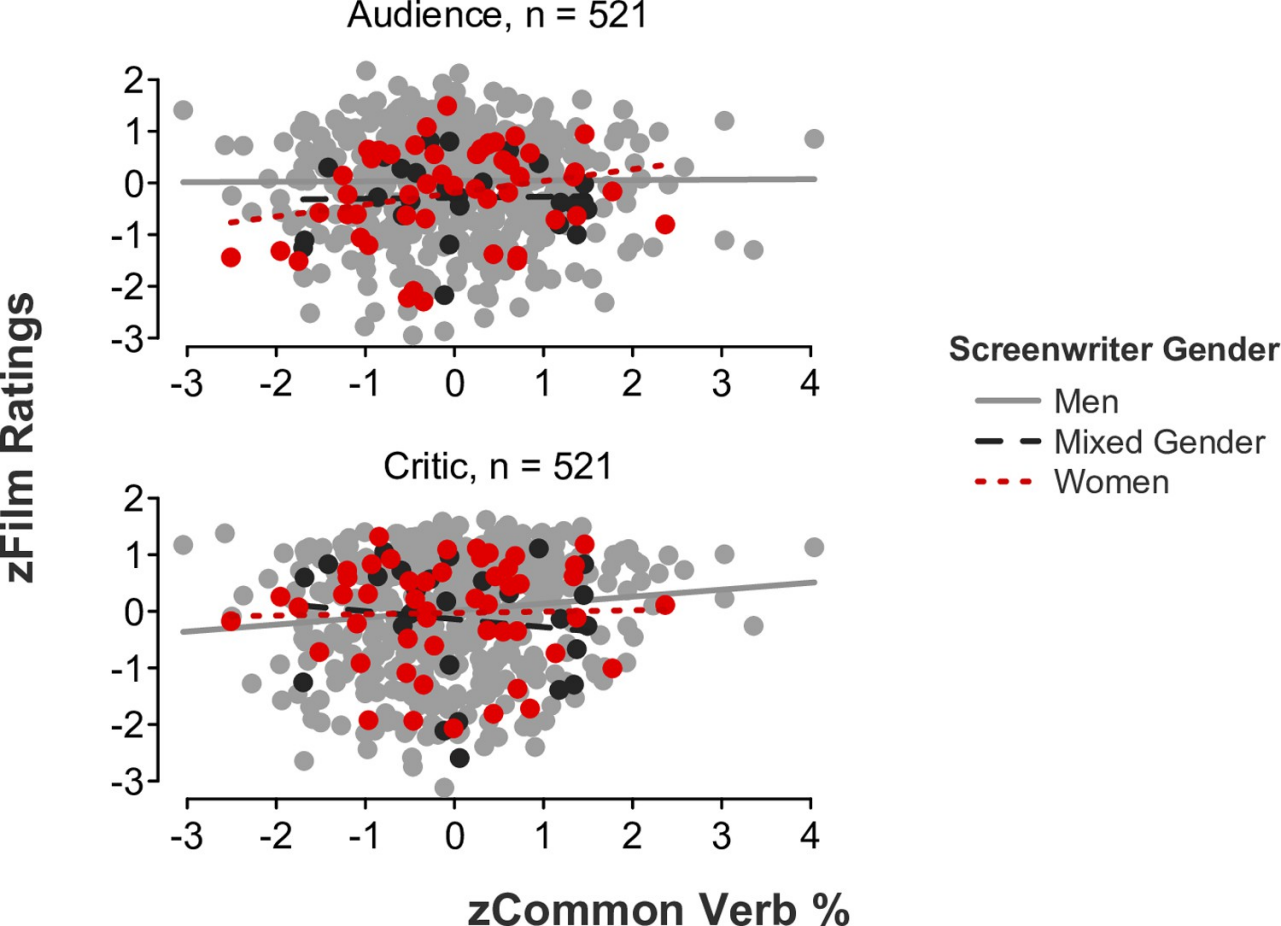
Film ratings as a function of common verb use, screenwriter gender, and rater role.

**Fig 4:**
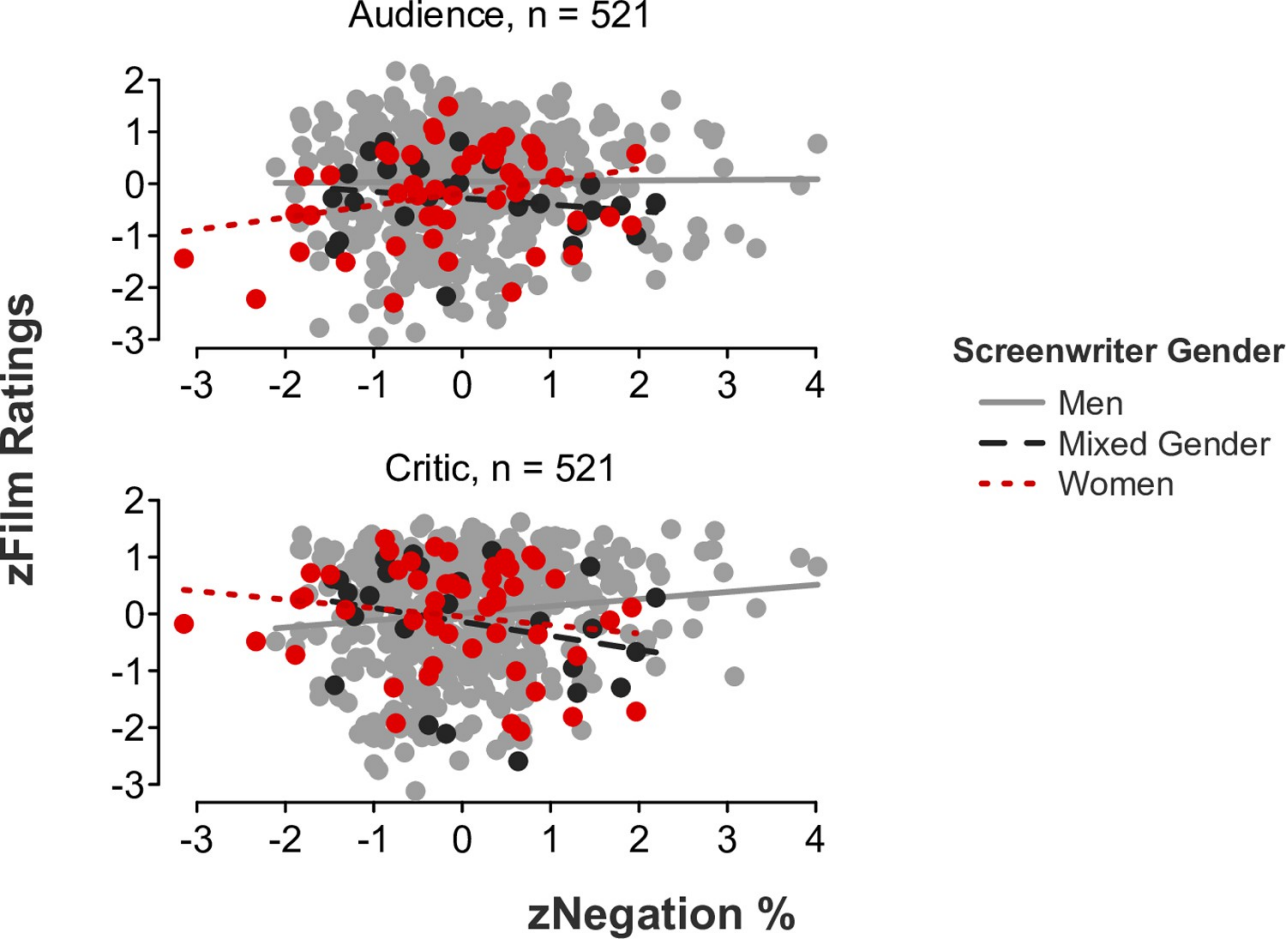
Film ratings as a function of negation use, screenwriter gender, and rater role.

*Feminine language*. Partially coinciding with our hypotheses, linear mixed-effects models showed significant *language* x *screenwriter gender* x *rater role* interaction effects for auxiliary verbs (*b* = -.29, *SE* = .10, *t*[515] = -2.84, *p* = .005, 95% CI [-0.50, -0.09]; Fig 2), common verbs (*b* = -.31, *SE* = .10, *t*[515] = -3.05, *p* = .002, 95% CI [-0.51, -0.11]; Fig 3), and negations (*b* = -.48, *SE* = .10, *t*[515] = -5.04, *p* < .001, 95% CI [-0.67, -0.30]; Fig 4). Simple slope tests illustrated that critics gave significantly higher ratings to films written by men who used a higher rate of auxiliary verbs (*b* = .15, *SE* = .05, *t*[443] = 3.12, *p* = .002, 95% CI [0.05, 0.24]), common verbs (*b* = .12, *SE* = .05, *t*[443] = 2.55, *p* = .011, 95% CI [0.03, 0.21]), and negations (*b* = .12, *SE* = .05, *t*[443] = 2.54, *p* = .012, 95% CI [0.03, 0.22]) in their scripts. In contrast, simple slope tests demonstrated that audiences gave significantly higher ratings to films written by women who used a higher rate of auxiliary verbs (*b* = .26, *SE* = .12, *t*[48] = 2.07, *p* = .044, 95% CI [0.01, 0.50]) in their scripts. A more modest trend was found for simple slopes examining common verbs and negations, wherein audiences gave slightly higher ratings to films written by women who used a higher rate of common verbs (*b* = .22, *SE* = .12, *t*[48] = 1.80, *p* = .078, 95% CI [-0.03, 0.47]) and negations (*b* = .23, *SE* = .12, *t*[48] = 1.98, *p* = .053, 95% CI [-0.003, 0.46]) in their scripts. All remaining simple slope tests were nonsignificant, all *p*s > .1.

There were no significant *language* x *screenwriter gender* x *rater role* interaction effects for third-person singular pronouns (*b* = -.16, *SE* = .11, *t*[515] = -1.46, *p* = .144, 95% CI [-0.38, 0.05]), first-person singular pronouns (*b* = -.21, *SE* = .11, *t*[515] = -1.93, *p* = .054, 95% CI [-0.43, 0.004]), adverbs (*b* = -.07, *SE* = .11, *t*[515] = -0.66, *p* = .506, 95% CI [-0.29, 0.14]), conjunctions (*b* = -.08, *SE* = .11, *t*[515] = -0.71, *p* = .478, 95% CI [-0.31, 0.14]), or social words (*b* = -.18, *SE* = .10, *t*[515] = -1.78, *p* = .076, 95% CI [-0.37, 0.02]).

**Fig 5:**
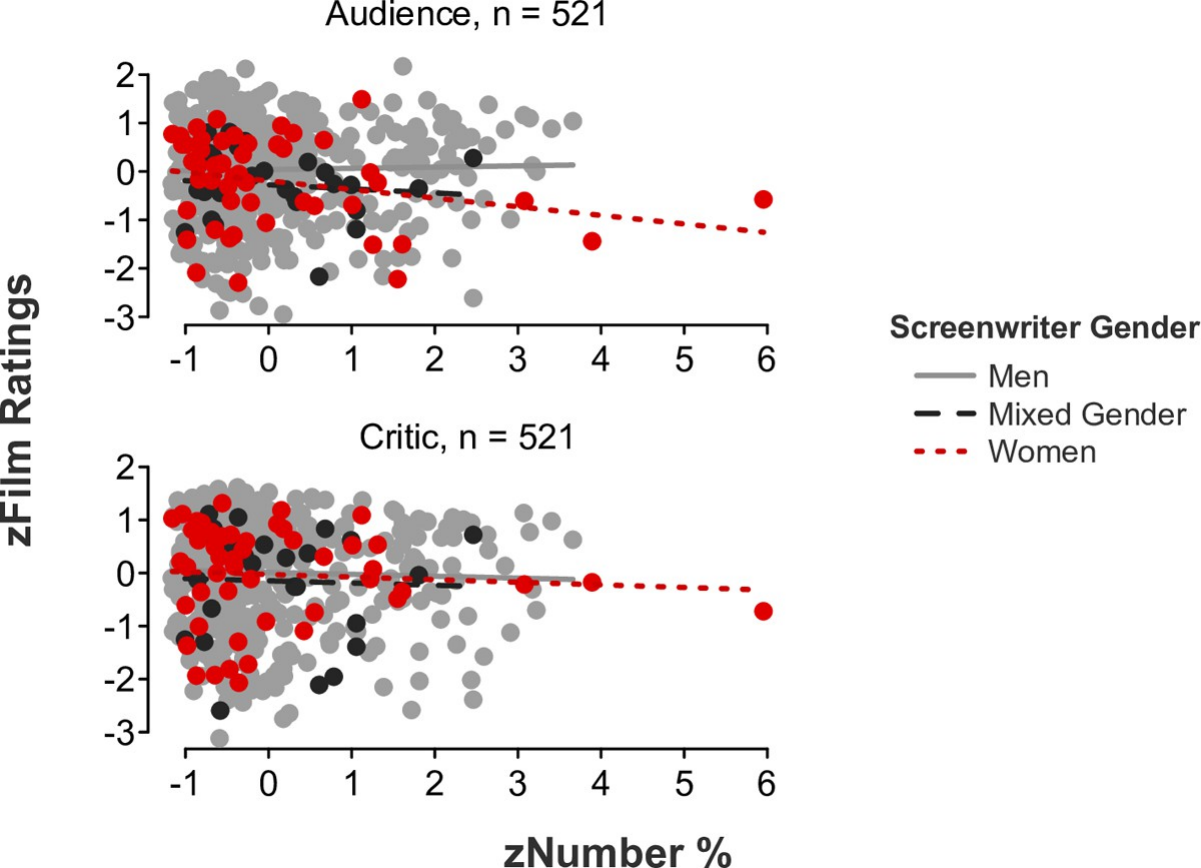
Film ratings as a function of use of numbers, screenwriter gender, and rater role.

*Masculine language*. Results partly supported our hypotheses regarding masculine language categories in film scripts. Linear mixed-effects models indicated a significant *language* x *screenwriter gender* x *rater role* interaction effect for numbers (*b* = .21, *SE* = .09, *t*[515] = 2.34, *p* = .020, 95% CI [0.03, 0.39]; Fig 5). Follow-up simple slope tests found a modest trend, wherein audiences gave slightly higher ratings to films written by women who used fewer numbers in their scripts (*b* = -.20, *SE* = .11, *t*[48] = -1.91, *p* = .063, 95% CI [-.042, 0.01]). Additional simple slope tests were nonsignificant, *p*s > .1.

Although there was a significant *language* x *screenwriter gender* x *rater role* interaction effect for swear words (*b* = -.21, *SE* = .10, *t*[515] = -2.04, *p* = .042, 95% CI [-0.41, -0.01]), follow-up simple slope tests were not significant (*p*s > .1). Further, there were no significant *language* x *screenwriter gender* x *rater role* interaction effects for words with more than six letters (*b* = -.02, *SE* = .09, *t*[515] = -0.20, *p* = .843, 95% CI [-0.19, 0.15]), quantifiers (*b* = .06, *SE* = .10, *t*[515] = 0.61, *p* = .540, 95% CI [-0.13, 0.25]), articles (*b* = -.03, *SE* = .12, *t*[515] = -0.25, *p* = .799, 95% CI [-0.26, 0.20]), or prepositions (*b* = .03, *SE* = .11, *t*[515] = 0.27, *p* = .790, 95% CI [-0.18, 0.24]).

#### Covariate analyses

In order to account for previous literature [11, 32] implicating genre as a potential confounding variable for effects of gender-linked language in film, we ran our original main and interaction effects models with the addition of genre as a control variable. We also added the topical language categories (work, leisure, home, religion, death, and money) to our original models as a way to further control for varying topics within film scripts. The main effect of screenwriter gender on the gender-linked language composite—previously nonsignificant—reached statistical significance when genre and the topical language categories were added to the model as covariates (*F*[2] = 3.90, *p* = .021, *η*_p_^2^ = .02). Follow-up Tukey HSD tests revealed that female screenwriters now significantly scored higher on the gender-linked language composite (i.e., used more feminine and less masculine language in their scripts) than male screenwriters (*p* = .018, 95% CI [0.05, 0.64]). All other significant main effects of screenwriter gender on language (i.e., articles, prepositions, third-person pronouns, and social words) remained significant after controlling for genre and the topical language categories, suggesting that the results are independent of film genre and topic.

Similarly, all previously significant *language* x *screenwriter gender* x *rater role* interaction effects remained significant after controlling for film genre and topic. However, for both audience and critic ratings, the majority of simple slope effects for the association between gender-linked language (individual categories or the composite score) and ratings were no longer significant (*p*s > .1) after controlling for film genre and topic. Only one simple slope effect retained its significance with the addition of the covariates; specifically, for critics’ ratings of films by male screenwriters, the positive association between auxiliary verb use and ratings remained significant. Nevertheless, because most of these simple slope effects were modest to begin with, they did not survive the addition of seven covariates and the consequently restricted degrees of freedom, especially considering the uneven sub-samples of film scripts written by men (*n* = 445) and women (*n* = 50). In fact, when conducting simple slope tests for each covariate separately, most models either remained significant (*p* < .05) or retained a modest, nonsignificant trend (*p* < .1). Overall, based on our results from both our main effect and interaction effect covariate models, film genre and topic do not appear to impact our findings regarding gender-linked language in film.

### Discussion

Consistent with past work on gendered language, our findings illustrate that female screenwriters use a feminine style of writing (higher rates of third-person singular pronouns and social words) and male screenwriters use a masculine style of writing (higher rates of articles and prepositions). We also found that audiences were more likely to prefer films by women using a feminine (gender-congruent) language style in their scripts, while professional film critics were more likely to prefer films by men using a feminine (gender-incongruent) language style in their scripts. Our findings generally held even after controlling for varying genres and topics of films.

Nevertheless, one of the main issues of Study 1 remains the limited number of film scripts written by women (*n* = 50) in the current sample. As previously noted, the film industry is fraught with gender disparities, wherein the prevalence of women in directorial or screenwriting positions is quite low [2, 3, 38–40]. Such real-life disparities are reflected in our own small sample of female screenwriters. In order to determine whether our initial findings can be generalized to other samples, we conducted a second study examining a sample of novels with an equal number of male and female novelists.

## Study 2

### Method

In Study 2, we conducted an archival analysis as a way to establish whether our findings from Study 1—concerning gender, language, and film ratings—would also extend to a sample of novels by men and women. Based on previous findings as well as our own results from Study 1, we hypothesized that novels by female writers would more likely exhibit a feminine language style, while novels by male writers would more likely exhibit a masculine writing style. Moreover, we predicted that reader ratings would be higher for novels with a writing style congruent with the author’s gender (i.e., higher ratings for novels by women employing a feminine writing style and for novels by men employing a masculine writing style). The data collection and sharing procedures of Study 2 are consistent with *PLOS ONE*’s data management and availability policies. The dataset used to conduct all descriptive and inferential statistical analyses highlighted in Study 2 (*Novel Data*) is publicly available on the Open Science Framework (OSF; see https://osf.io/jgcnu/).

#### Sample

We collected 150 novels from Project Gutenberg (see www.gutenberg.org), a website that contains more than 60,000 ebooks that are free to the public. The year of publication for novels in the present sample ranged from 1666 to 1957. We obtained an equal number of novels written by men and women (75 novels by men and 75 novels by women). Novels were included in the current sample if (1) a link of the full text of the novel could be found on Project Gutenburg, and (2) at least 20 users rated the novel on GoodReads (see www.goodreads.com), the website we used to measure audience ratings of novels. The data collected from Project Gutenburg and GoodReads are publicly available, and the collection method complied with the terms and conditions for each website.

#### Measures

*Linguistic inquiry and word count*. Once again, LIWC [41] was used to measure the frequency (total %) of gender-linked language categories within novels. The same language categories analyzed in Study 1 were also analyzed in Study 2. For our main analyses, feminine (or female-linked) language consisted of first- and third-person singular pronouns, common verbs, adverbs, auxiliary verbs, conjunctions, negations, and social words, while masculine (or male-linked) language consisted of words with more than six letters, numbers, quantifiers, articles, prepositions, and swear words (see Table 1 for examples). First-person singular pronouns, numbers, quantifiers, and swear words were all positively skewed. First-person singular pronouns and numbers were both transformed using the square root transformation, while quantifiers necessitated the log base 10 transformation to reach normality. Transformations did not help with the positive skew of swear words, even after winsorizing the one outlier—*Ragged Dick* by Horatio Alger Jr.—most likely due to the extremely low base rate of swear words in our sample of novels (*M* = .04, *SD* = .07). Thus, swear words was left untransformed. Social language was negatively skewed and transformed by squaring the variable. All gender-linked language categories were standardized (z-scored) and used to compute the gender-linked language composite also calculated in Study 1 (see Eq 1).

Additionally, the topical language categories analyzed in Study 1 as covariates were added to the main statistical models in Study 2: work, home, death, religion, leisure, and money (refer to Table 1). Home words were normally distributed; however, work, death, religion, leisure, and money were all positively skewed and subsequently transformed using the log base 10 transformation. All topical language covariates were standardized.

*Novel ratings*. Audience ratings for novels were obtained from GoodReads (https://www.goodreads.com/), a website dedicated to helping readers find their next book. GoodReads allows users of the site to rate books on a numerical scale out of 5 stars. The GoodReads rating was negatively skewed and was transformed (and standardized) by cubing the variable to resemble a more normal curve.

Unlike Study 1, we did not consider critics’ reviews in comparison with readers’ reviews. The novels analyzed in this study ranged widely in popularity and year of publication; thus, many novels lacked a sufficient sample (e.g., *N* > ~ 20) of professional reviews available online, and all novels were in the public domain. Therefore, the novel sample lacked the kind of curated, contemporaneous reviews that made the ratings aggregated on Rotten Tomatoes so psychometrically attractive and tractable.

#### Statistical analyses

To test the main effect of novelist gender on language, we ran Welch’s independent samples *t*-tests for the gender-linked language composite as well as for each of the language categories that make up the composite. In order to test the interaction effect of novelist gender and language correlating with novel ratings, we ran multiple regression models for the gender-linked language composite and each language category within the composite. More specifically, we regressed novel ratings on novelist gender for each language category (e.g., Novel Ratings ~ Language Category * Novelist Gender). *Novel Ratings* (outcome variable) and *Language Category* (explanatory variable) were both treated as continuous variables, and *Novelist Gender* was treated as a categorical (moderator) variable.

Topical language categories (work, home, death, religion, leisure, and money) were also added to both main effect and interaction effect models to control for topics within novels. For main effects of novelist gender on language, we conducted ANCOVAs by adding all topic covariates to the model: *Language Category ~ Novelist Gender + Work + Home + Death + Religion + Leisure + Money*. For interaction effects of novelist gender and language on novel ratings, we conducted multiple regression models with the addition of the topic covariates: Novel Ratings ~ Language Category * Novelist Gender + Work + Home + Death + Religion + Leisure + Money. All analyses were conducted in R [43].

Similar to Study 1, we reported several *t*-tests for each gender-linked language category rather than a single MANOVA examining the linear combination of all categories with the rationale that: (1) each linguistic variable characterizes a different feature of gender and (2) the MANOVA was statistically significant (*p* < .001) with follow-up discriminant analyses illustrating that the strongest linguistic predictors of author gender were identical to those yielding significant results in the *t*-tests. Likewise, multiple regression models were reported for the interaction effects of author gender and language category on novel ratings rather than a two-way MANOVA because MANOVAs require categorical independent variables and our language variables are continuous.

### Results

#### Gender differences in novels

Coinciding with previous research on gender-linked language as well as our Study 1 findings on screenwriter gender and language in film scripts, an independent samples *t*-test revealed that female authors scored significantly higher on the gender-linked language composite (i.e., more feminine and less masculine language in novels) than male authors (*t*(140.9) = -4.53, *p* < .001, 95% CI [-1.00, -0.39]). However, to determine whether specific gender-linked language categories were driving the effect of novelist gender, we also conducted independent samples *t*-tests for each of the language categories that make up the gender-linked language composite.

Consistent with past work and our own screenwriter gender and language results, independent samples *t*-tests exhibited significant main effects of novelist gender on the following feminine language categories: third-person singular pronouns (*t*[146.5] = -2.28, *p* = .024, 95% CI [-0.69, -0.05]), negations (*t*[147.3] = -3.60, *p* < .001, 95% CI [-0.88, -0.26]), and social words (*t*[147.7] = -4.17, *p* < .001, 95% CI [-0.95, -0.34]). Modest trends (*p* < .1) of novelist gender on female-linked language were found for common verbs (*t*[147.3] = -1.88, *p* = .062, 95% CI [-0.62, 0.02]) and auxiliary verbs (*t*[142.9] = -1.75, *p* = .082, 95% CI [-0.60, 0.04]). Specifically, female novelists used significantly more third-person singular pronouns, negations and social words—as well as slightly more common verbs and auxiliary verbs—than male novelists. Main effects of first-person singular pronouns, adverbs, and conjunctions were not statistically significant (all *p*s > .1).

Complementing our novelist gender and feminine language results, independent samples *t*-tests demonstrated significant main effects of novelist gender on the following masculine language categories: articles (*t*[142.9] = 5.42, *p* < .001, 95% CI [0.51, 1.11]), prepositions (*t*[146.9] = 2.82, *p* = .006, 95% CI [0.13, 0.77]), numbers (*t*[147.1] = 5.01, *p* < .001, 95% CI [0.46, 1.06]), and swear words (*t*[87.1] = 3.03, *p* = .003, 95% CI [0.17, 0.80]). Particularly, male novelists used significantly more articles, prepositions, numbers, and swear words than female novelists. Main effects of quantifiers and words made up of more than six letters were not statistically significant (all *p*s > .1). See Table 3 for specific mean differences in language use between male and female novelists.

**Table 3:** Mean differences of language use in novels across male and female authors.

	Female	Male
	*M* (*SD*)	*M* (*SD*)
*Gender-Linked Language Composite* *	0.35 (0.83)	-0.35 (1.04)
*Feminine Language*		
I	3.02 (1.60)	2.90 (1.86)
SheHe*	5.29 (1.74)	4.68 (1.57)
Verbs†	15.28 (1.96)	14.66 (2.10)
Adverbs	4.33 (0.59)	4.25 (0.63)
Auxiliary Verbs†	7.69 (1.07)	7.35 (1.30)
Conjunctions	6.94 (0.91)	6.74 (1.07)
Negations*	1.72 (0.39)	1.50 (0.36)
Social*	13.37 (1.99)	11.85 (2.43)
*Masculine Language*		
Numbers*	0.98 (0.30)	1.24 (0.35)
> Six Letters	16.29 (2.90)	16.11 (2.74)
Quantifiers	1.99 (0.42)	1.94 (0.36)
Articles*	7.23 (1.09)	8.30 (1.32)
Prepositions*	14.12 (1.01)	14.61 (1.11)
Swear*	0.02 (0.03)	0.06 (0.09)

With the exception of the gender-linked language composite, the above means and standard deviations are derived from the non-standardized language categories. All numbers are percentages obtained from LIWC [41].

*p < .05.

†*p* < .1.

#### Audience ratings of novels

**Fig 6:**
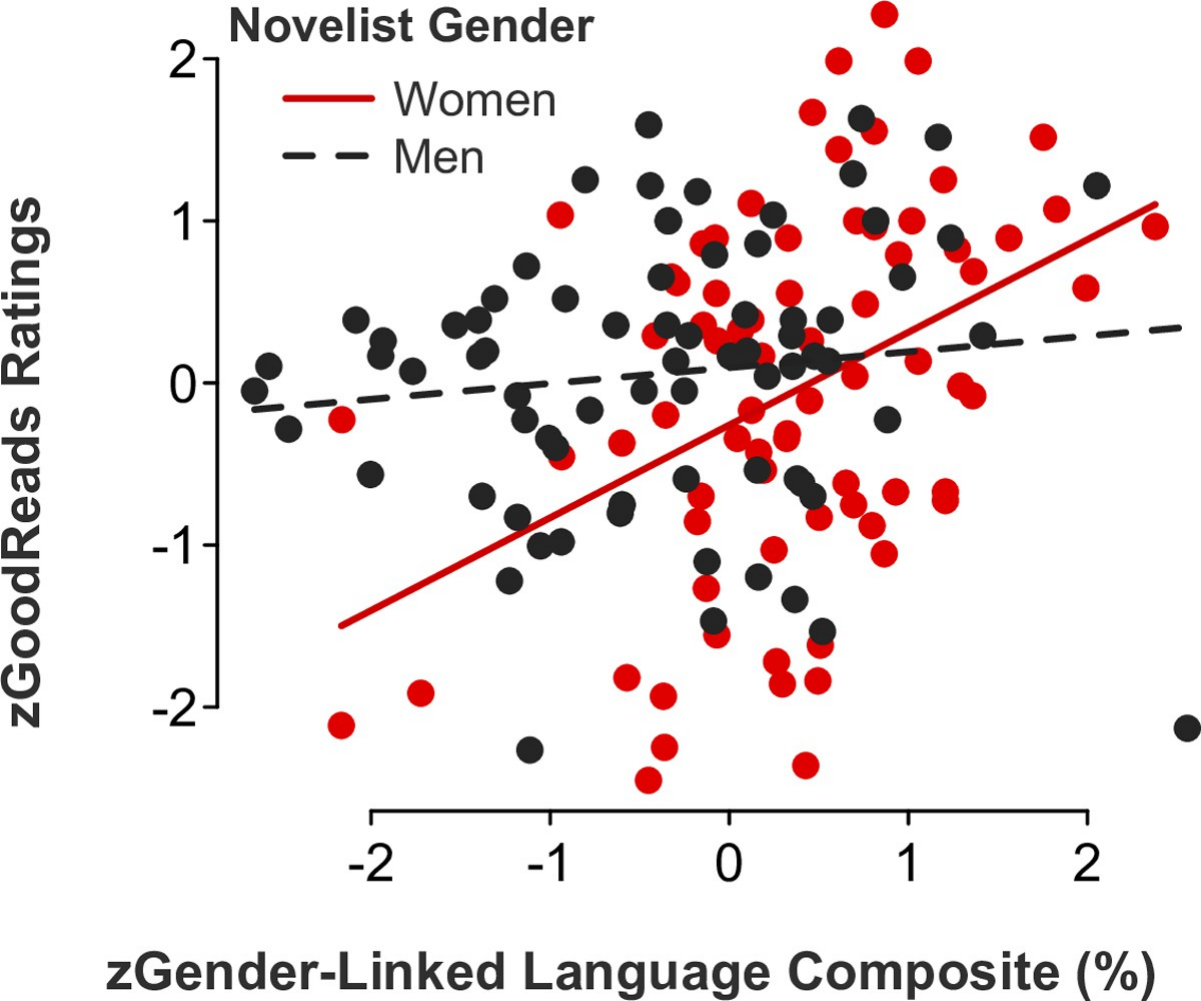
Novel ratings as a function of the gender-linked language composite and novelist gender.

Based on our Study 1 findings (demonstrating how audiences favored films by female screenwriters incorporating more female-linked and less male-linked language in their scripts), we predicted that readers would give higher ratings to novels by authors using a gender-congruent language style in their work. Consistent with the pattern of findings from Study 1, the multiple regression model examining GoodReads novel ratings as a function of the gender-linked language composite and author gender was statistically significant (*b* = 0.47, *SE* = .17, *t*[146] = 2.79, *p* = .006, 95% CI [0.14, 0.81]; Fig 6). Follow-up simple slope tests further corroborated that readers gave significantly higher ratings to novels by women (but not men, *p* > .1) using more feminine and less masculine language (*b* = 0.57, *SE* = .15, *t*[73] = 3.92, *p* < .001, 95% CI [0.28, 0.86]).

**Fig 7:**
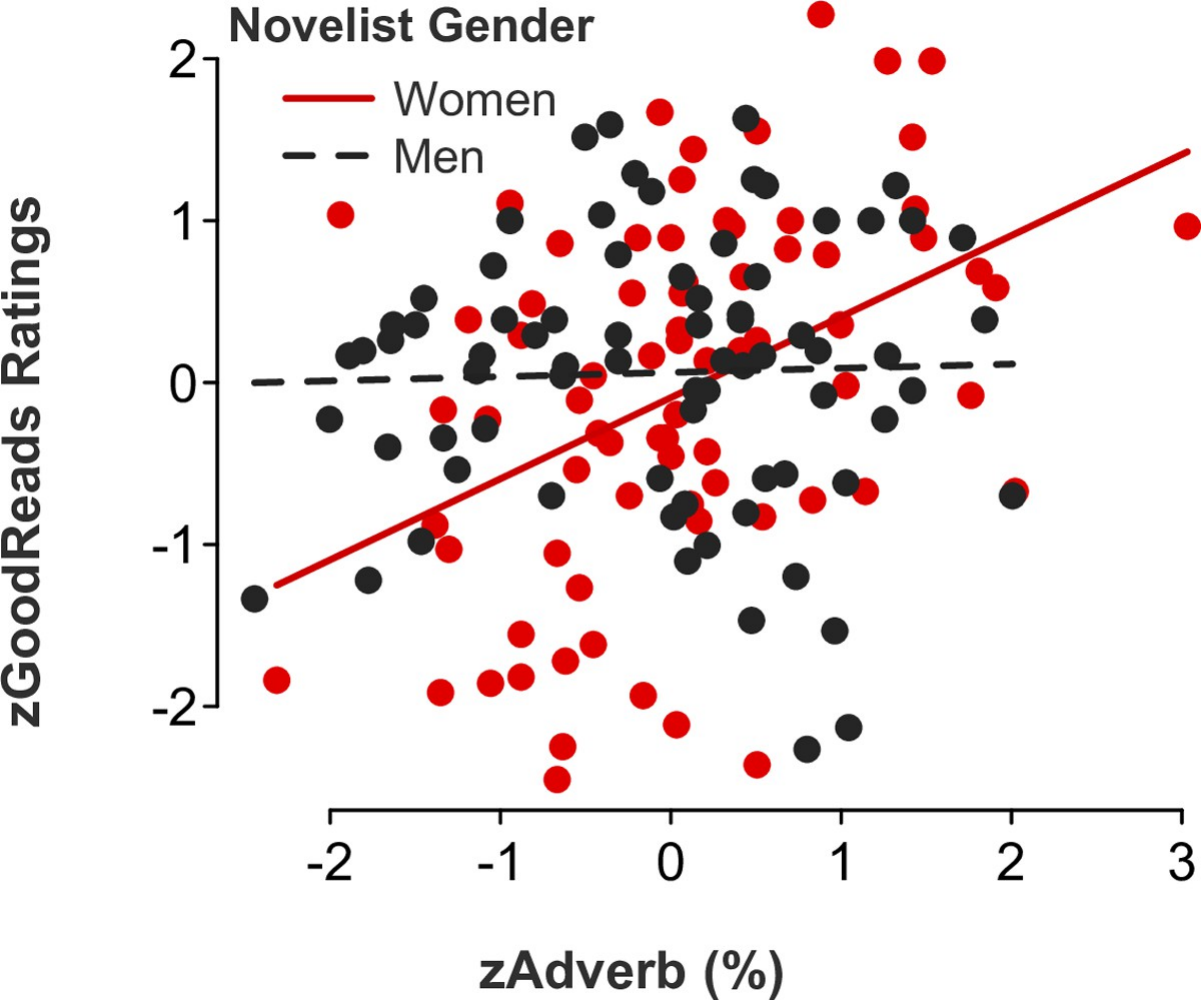
Novel ratings as a function of adverb use and novelist gender.

**Fig 8:**
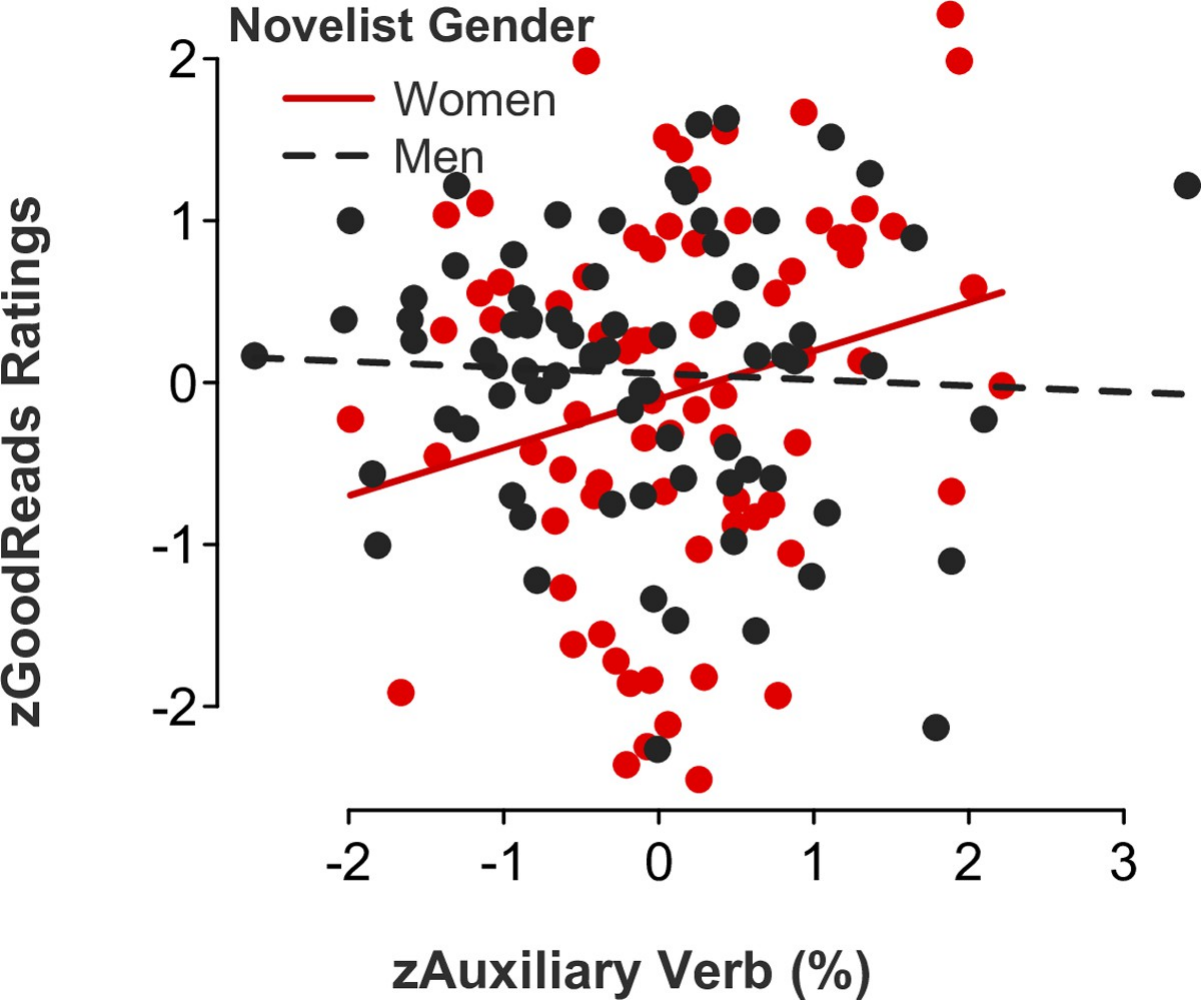
Novel ratings as a function of auxiliary verb use and novelist gender.

**Fig 9:**
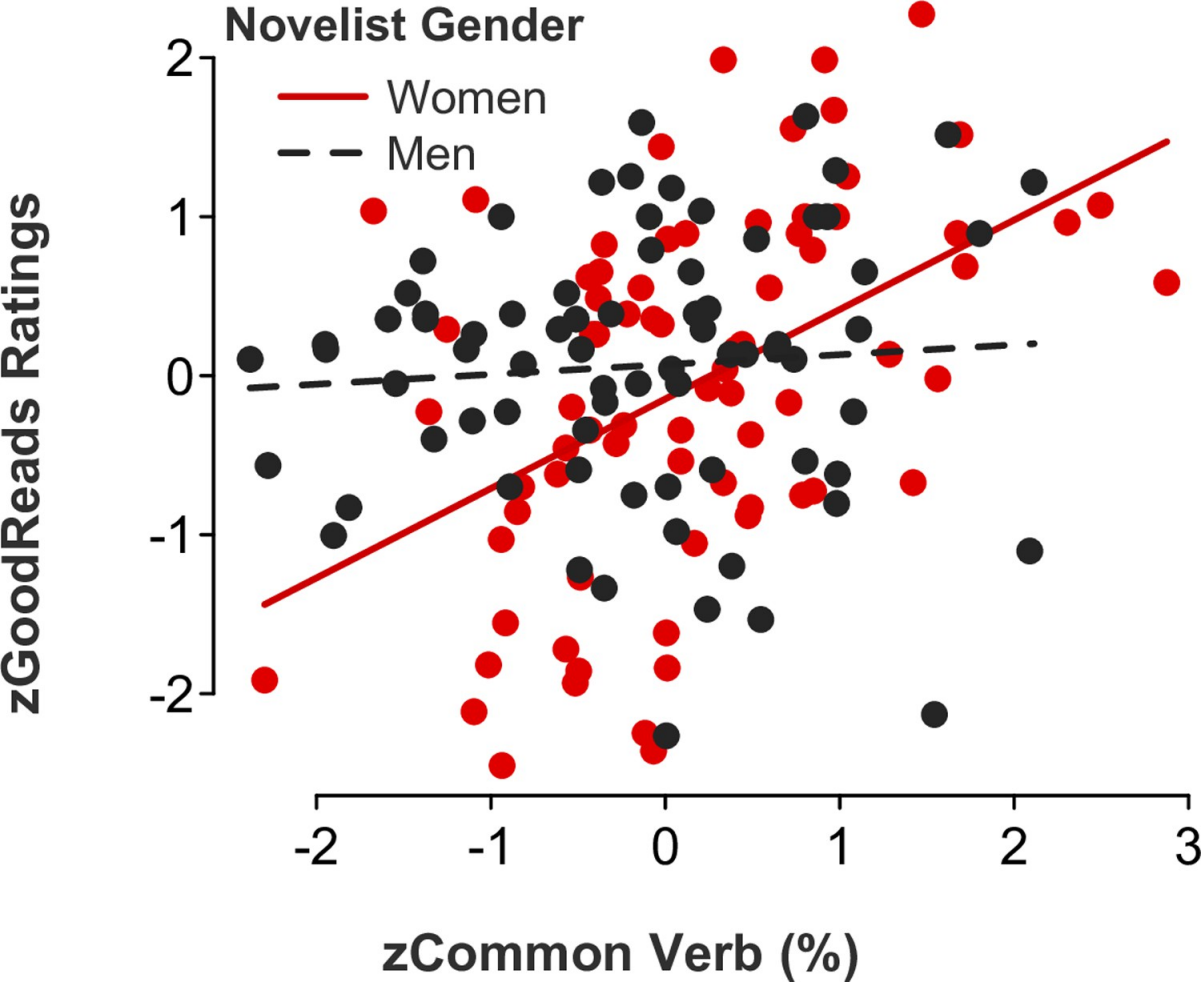
Novel ratings as a function of common verb use and novelist gender.

**Fig 10:**
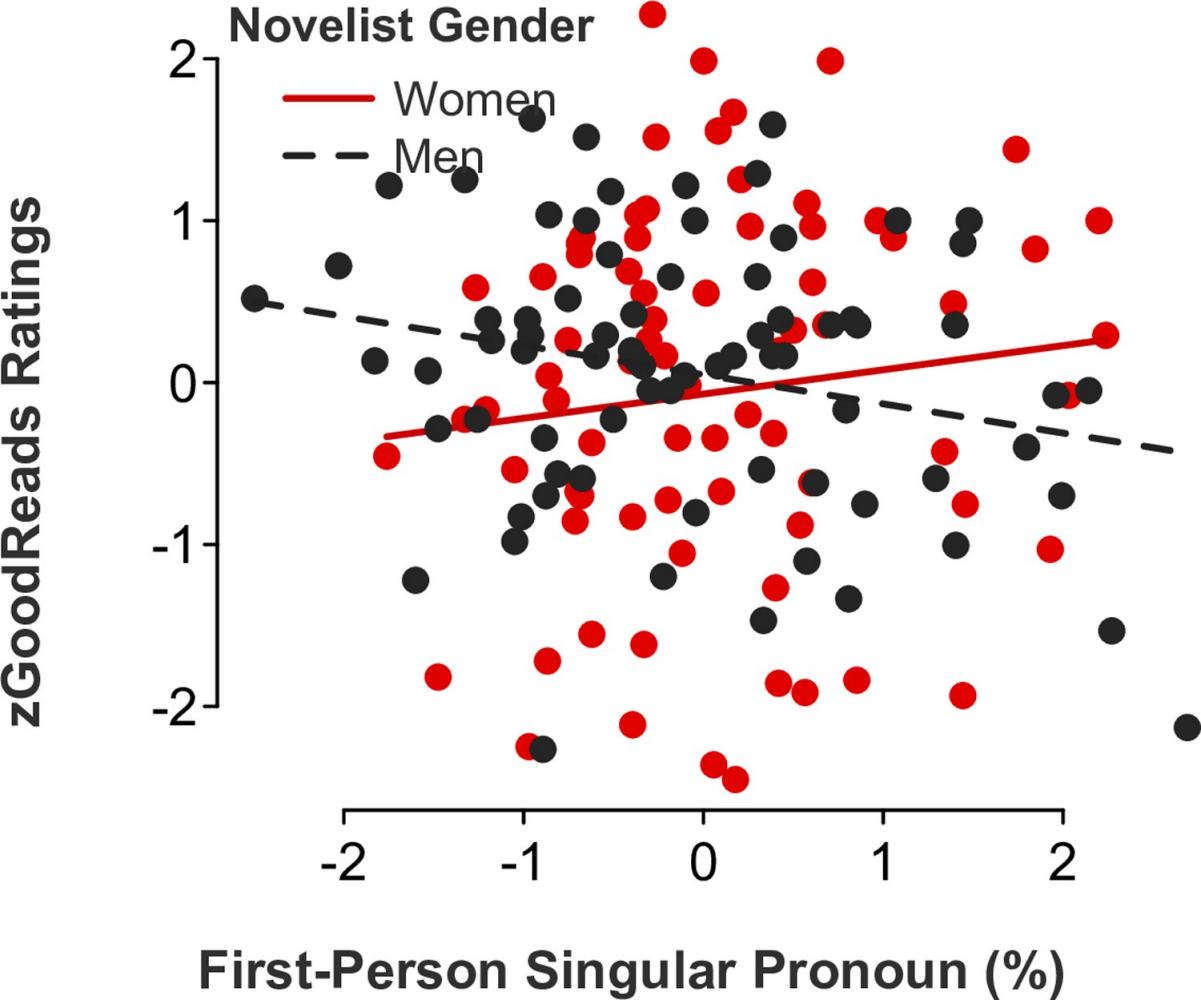
Novel ratings as a function of first-person singular pronoun use and novelist gender.

*Feminine language*. Partly consistent with our Study 1 results examining film ratings as a function of screenwriter gender, gender-linked language, and rater role (audience versus critic ratings), multiple regression models showed significant *novelist gender x feminine language* interaction effects for the following categories: adverbs (*b* = 0.47, *SE* = .16, *t*[146] = 3.05, *p* = .003, 95% CI [0.17, 0.78]; Fig 7), auxiliary verbs (*b* = 0.34, *SE* = .17, *t*[146] = 2.01, *p* = .046, 95% CI [0.01, 0.67]; Fig 8), common verbs (*b* = 0.50, *SE* = .15, *t*[146] = 3.24, *p* = .002, 95% CI [0.19, 0.81]; Fig 9), and first-person singular pronouns (*b* = 0.33, *SE* = .17, *t*[146] = 1.98, *p* = .049, 95% CI [0.001, 0.66]; Fig 10). Follow-up simple slope tests illustrated that readers gave significantly higher ratings to novels by women (but not men, *p* > .1) who used higher rates of adverbs (*b* = 0.50, *SE* = .12, *t*[73] = 4.04, *p* < .001, 95% CI [0.25, 0.75]), auxiliary verbs (*b* = 0.30, *SE* = .14, *t*[73] = 2.06, *p* = .043, 95% CI [0.01, 0.59]), and common verbs (*b* = 0.56, *SE* = .12, *t*[73] = 4.59, *p* < .001, 95% CI [0.32, 0.81]). Additional simple slope tests demonstrated that readers gave significantly higher ratings to novels by men (not women, *p* > .1) who used lower rates of first-person singular pronouns (*b* = -0.18, *SE* = .09, *t*[73] = -2.05, *p* = .044, 95% CI [-0.36, -0.01]). There were no statistically significant *novelist gender x feminine language* interaction effects for third-person singular pronouns (*b* = -0.24, *SE* = .17, *t*[146] = -1.46, *p* = .145, 95% CI [-0.57, 0.09]), conjunctions (*b* = 0.06, *SE* = .17, *t*[146] = 0.38, *p* = .700, 95% CI [-0.27, 0.40]), negations (*b* = 0.17, *SE* = .17, *t*[146] = 1.02, *p* = .311, 95% CI [-0.16, 0.51]), or social words (*b* = -0.19, *SE* = .17, *t*[146] = -1.06, *p* = .289, 95% CI [-0.53, 0.16]).

**Fig 11:**
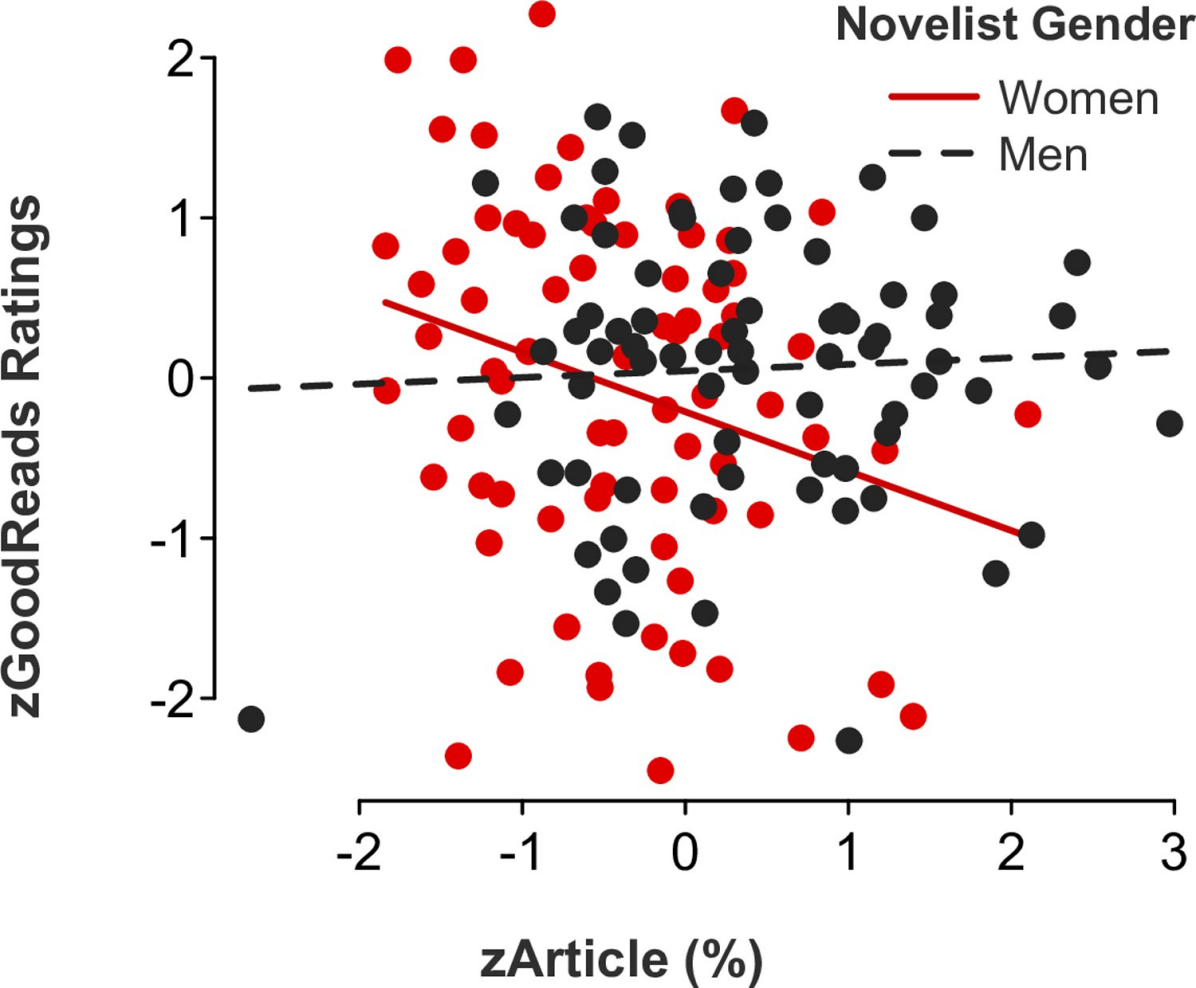
Novel ratings as a function of article use and novelist gender.

*Masculine language*. Somewhat consistent with the results of Study 1, a multiple regression model displayed a significant *novelist gender x masculine language* interaction effect for articles (*b* = -0.41, *SE* = .18, *t*[146] = -2.29, *p* = .023, 95% CI [-0.77, -0.06]; Fig 11). Follow-up simple slope tests showed that readers gave significantly higher ratings to novels by women (but not men, *p* > .1) who used lower rates of articles (*b* = -0.37, *SE* = .16, *t*[73] = -2.39, *p* = .020, 95% CI [-0.68, -0.06]). There were no statistically significant *novelist gender x masculine language* interaction effects for words with more than six letters (*b* = -0.29, *SE* = .16, *t*[146] = -1.86, *p* = .065, 95% CI [-0.59, 0.02]), prepositions (*b* = -0.23, *SE* = .17, *t*[146] = -1.41, *p* = .161, 95% CI [-0.56, 0.09]), quantifiers (*b* = -0.06, *SE* = .16, *t*[146] = -0.40, *p* = .694, 95% CI [-0.38, 0.25]), numbers (*b* = 0.12, *SE* = .18, *t*[146] = 0.69, *p* = .493, 95% CI [-0.23, 0.47]), or swear words (*b* = 0.44, *SE* = .31, *t*[146] = 1.43, *p* = .154, 95% CI [-0.17, 1.05]).

#### Covariate analyses

Overall, the main effect models testing gender-linked language differences between male and female novelists revealed that female authors incorporated more female-linked words and fewer male-linked words in their novels than male authors. However, to further substantiate this pattern of findings and confirm whether they are confounded by varying topics illustrated in the sample of novels, we ran our original main effect models with the addition of topical language categories serving as covariates. Subsequent ANCOVAs demonstrated that our original significant findings remained significant after controlling for work, home, death, religion, leisure, and money language in novels. Thus, novelist gender holds a significant effect on the gender-linked language composite (and, specifically, third-person singular pronouns, negations, social words, articles, prepositions, numbers and swear words) above and beyond differing topics portrayed in novels.

Earlier we found that readers gave more positive ratings to novels written by authors who adhered to a gender-congruent style of writing (e.g., female novelists who used higher rates of female-linked language and lower rates of male-linked language). In order to explore whether those effects are attributable to gender-linked writing styles being confounded by narrative topics, we added the same topical language categories from our main effect covariate models to our interaction effect models. When controlling for work, home, death, religion, leisure, and money language, most *novelist gender x language* interaction effects and follow-up simple slope effects that were previously significant (i.e., models including the gender-linked language composite, adverbs, common verbs, and articles) remained significant. Models containing auxiliary verbs and first-person singular pronouns—which were originally modest effects—were not sustained with the addition of the six covariates. However, when analyzing the interaction and simple slope effects for those models with each covariate separately, most results retained significance (*p* < .05) or a modest trend (*p* < .1). Therefore, the covariate models further establish that the main and interaction effects hold true regardless of different topics presented within novels.

### Discussion

The purpose of Study 2 was to replicate our findings from Study 1 and determine whether gender differences between male and female screenwriters and the associations between gendered language and audience ratings would extend to a sample of novels. The results in Study 1 were mostly reproduced in Study 2. Female novelists were more likely to use female-linked (or feminine) language in their novels, whereas male novelists were more likely to use male-linked (or masculine) language in their novels. Readers also rated novels by female authors more positively if they incorporated more feminine language and less masculine language in their writing; in contrast, readers rated novels by male authors more positively if they incorporated less feminine (i.e., more masculine) language. Overall, our findings remained significant regardless of topic. Thus, complementing our Study 1 findings, audiences gave higher ratings to narratives that use language that is more congruent with the writer’s gender.

## General discussion

A computerized text analysis of language used in popular films scripts and classic novels revealed that both screenwriters and novelists tend to follow gender-linked patterns of writing. Female screenwriters and novelists used more feminine language in their narratives than their male counterparts. In particular, female writers in both mediums incorporated more third-person singular pronouns (e.g., *she*, *him*) and social words (e.g., *friend*, *chat*) than male writers; female novelists additionally used more negations (e.g., *no*, *doesn’t*), auxiliary verbs (e.g., *might’ve*, *become*), and common verbs (e.g., *leave*, *playing*) in their writing, relative to male novelists. On the other hand, male screenwriters and novelists used more masculine language in their narratives than their female counterparts. Specifically, male writers in both mediums included more articles (e.g., *a*, *the*), prepositions (e.g., *on*, *about*), and swear words (e.g., *damn*, *hell*) in their work, in addition to higher rates of numbers (e.g., *twice*, *zillion*) for male novelists, relative to female writers. These gender differences in language are consistent with findings across decades of research demonstrating the potential existence of either biological or societal factors—or some combination of the two—that account for these differences.

Results across the two studies generally coincided with our hypotheses regarding narrative reception as well. Audiences and readers appeared to enjoy films and novels more to the degree that the writer adhered to a gender-congruent language style, whereas professional critics demonstrated the opposite pattern for films. More specifically, audiences and readers gave higher ratings to films and novels written by women that were consistent with a feminine language style (higher rate of auxiliary verbs, common verbs, and negations for films and auxiliary verbs, common verbs, and adverbs for novels) and inconsistent with a masculine writing style (lower rate of numbers for films and articles for novels). Readers also gave higher ratings to novels by men that were consistent with a masculine writing style (i.e., lower rates of first-person singular pronouns). Finally, professional film critics gave higher ratings to films written by men that were consistent with a feminine pattern of writing (again, higher rate of auxiliary verbs, common verbs, and negations).

Nevertheless, our findings are correlational, and we are merely observing how audiences’ and critics’ receptions of narratives relate to gender-linked language patterns in scripts and novels by men and women. Experimental research is necessary to further unpack whether audiences and readers truly favor scripts and novels that conform to linguistic gender norms (with writers using gender-congruent language) and whether professional critics favor deviations from said norms (with writers using gender-incongruent language). For instance, audiences’ and readers’ preference for films and novels incorporating gender-congruent language may stem from theories of processing fluency (i.e., what is easier to understand and comprehend is easier to enjoy; [33]), unlike professional critics who might favor less typical (and thus more challenging or surprising) stimuli evidencing artistic creativity [35].

An alternative interpretation of our results is that rather than preferring gender-incongruent language, film critics may prefer language that is typical for the medium or for language use in general. In other words, perhaps they prefer gender-linked language that is average and thus androgynous, in a sense, rather than preferring gender-incongruent language per se. To test for this possibility, we averaged across all films in our sample, written by people of all genders, and correlated that average language profile with each film’s profile for the 14 gender-linked language categories used in our current study. After transforming those profile correlations to Fisher’s *z*, we regressed film ratings on the interaction of this new typicality score and rater category (film critic or audience). Results showed that audience members but not critics reviewed films more positively to the degree that the script’s language use was typical for the overall medium (all films sampled), suggesting that our previous effects were not attributable to film critics preferring average or typical language overall, irrespective of gender-congruence or incongruence.

Nevertheless, a few interaction effects between writer gender and masculine (e.g., words with more than six letters, quantifiers, prepositions, swear words) as well as feminine (e.g., conjunctions, third-person singular pronouns, social words) language categories—in both film scripts and novels—were nonsignificant. The nonsignificant findings might be explained by the implicit nature of function words and the explicit nature of content words. Content words (e.g., nouns, common verbs, adjectives) express *what* individuals discuss; function words (e.g., pronouns, prepositions, articles) define *how* those topics are discussed [9]. Function words—more so than content words—are used automatically by individuals, as they are predominantly short, high-frequency words that have little meaning outside of the context of a conversation or narrative [46]. Although film-viewing audiences as well as readers may attend to function words containing associative meaning (e.g., pronouns like *she* and *us* that are frequently used in reference to characters), other function words (e.g., articles like *a* and *the*, and prepositions like *on* and *to*) may simply receive limited attention and have little to do with audiences’ and readers’ experiences of narratives. Articles and prepositions were function word categories for which we found inconsistent (or null) rating-by-writer gender effects across studies; although we are wary of interpreting null results, those words may not have much effect on viewer and reader experiences at any level of awareness. That is, articles and prepositions may be less salient and influential than word categories that showed more consistent effects. Common verbs, for example, demonstrated robust rating-by-writer gender effects across both novels and films; verbs are content words that arguably carry more independent meaning than other function word categories like articles or prepositions. As a result, verbs may be more salient and garner more conscious attention in reading and conversation than other language categories.

We additionally considered that some of the results in both studies—null and significant—may have been partly influenced by the broad genres (e.g., romance or science fiction) and topics (e.g., death or leisure) that novels and films fell into. Genre often assists audiences and readers when choosing what films to watch or what books to read, and audience and reader ratings may be influenced by whether specific language within the narrative is consistent with its genre. Past research [37] illustrates that both lay audiences and professional critics are more likely to highly rate films with genre-typical language. Thus, if certain gender-linked language categories used in the present analyses are typical of one genre or another, that may skew the results. Social language—for example—is associated with women in stereotypes and, to some degree, reality. That is, the widely believed stereotype that women are more empathic or more concerned with social relationships has a modest kernel of truth, as found in experiments and survey-research on empathy [47] and linguistic analyses of natural language use [26, 27]. Individuals may then be more likely to consciously associate social words, and fictional genres focusing on social relationships (e.g., romance), with women [48] and may also prefer narratives with language that coincides with its genre. However, when controlling for genre and topic in our studies, most findings that were originally statistically significant remained significant or continued to show a modest trend in the original direction, suggesting that the interaction between gender-linked language and writer gender is independent of genre and topic.

Other mechanisms that might more readily influence narrative ratings and account for some of our results are audience or reader characteristics. Namely, in Study 1, we examined audience film ratings obtained from two sites: Rotten Tomatoes and IMDb. The culture surrounding these two popular film sites differ drastically. IMDb users are predominantly male [42] and—unlike Rotten Tomatoes—the site may not always censor trolls who purposefully rate films negatively based on their own political agenda (e.g., the alt-right group who orchestrated a virtual attack on audience ratings of *Black Panther*; [49]). We ran secondary analyses to determine whether the source of audience film ratings (Rotten Tomatoes versus IMDb) could potentially moderate our screenwriter gender and gender-linked language results and found no reliable interaction effects or differences between the sites’ simple slopes that could inform our current conclusions.

Although we did not have access to audience gender in either of our studies, the reviewer’s gender no doubt has relevance to the relations between gender-linked language, writer gender, and narrative ratings. In fact, qualitative research on IMDb audience reviews suggests that, oftentimes, audiences are consciously taking into account their own gender (among other demographic traits) when assessing a film [42]. For instance, in reference to the film *The Hangover*, one IMDb user remarked, “As a 32 year old all American white male, I should have thoroughly enjoyed this movie” [42], illustrating one of the many ways in which social roles and identity may influence judgments about what narratives individuals expect to enjoy. More relevant to the present research, audiences and readers who are themselves gender-typical may unconsciously recognize their own language style in the films they watch or the books they read by same-gender writers. Such language style matching—or verbal mimicry—has been linked with social engagement and relationship quality in past research [50]; thus, consuming fiction that matches a person’s own language style may on some level feel like an engaging conversation with a friend. Future research is needed to establish how audience characteristics such as gender, social identity, and individual differences in language style relate to the associations between gender-linked language, writer gender, and narrative preferences.

In addition to audience characteristics, an unknown factor that may have influenced audiences’ and readers’ preference for gender-congruent language and critics’ preference for gender-incongruent language could be knowledge or awareness of the gender of the writer. Film critics are bound by the norms of their profession to know something about the screenwriters behind the films they review. While readers are often aware of their novelist’s gender (since author names are readily obvious on the front cover of books), moviegoers may not attend to the opening credits of a film or research movies prior to viewing, particularly if they are more motivated by entertainment than by assessment purposes. If critics are consciously aware of the gender of a screenwriter for the film they are evaluating, they could be actively searching for deviations from gender norms in a script. However, audiences—who are likely less consciously aware of the screenwriter’s gender than critics or readers—may still be able to discern characteristics of a screenwriter based on the language used in the script.

For example, when presented with descriptive passages written by male or female college students, participants rated female writers higher in socio-intellectual status and aesthetic quality and male writers higher in dynamism, even without knowing the gender of the writer [36]. Furthermore, individuals are able to accurately guess the gender of an unknown e-mail correspondent just by reading their message [10]. That is, without conscious knowledge of the name or biography of the scriptwriter behind a film, audiences may be implicitly aware of the author’s likely gender as a result of overt (language content or topic; for example, films about weddings are more often written by women) or subtle (language style; for example, pronouns and adverbs) cues. In fact, our results demonstrated stronger patterns for reader ratings in Study 2 than for audience ratings in Study 1, suggesting that these effects are stronger with more certain or salient knowledge of the writer’s gender. Nevertheless, the degree to which audience members and professional critics are implicitly or explicitly aware of authors’ gender while viewing films, and the impact of that awareness on their viewing experience, remains to be assessed in future research.

The present research is also limited by its correlational nature and reliance on archival samples. Although archival studies provide valuable naturalistic data, correlational research of any kind cannot assume causality—thus, we can only speculate that certain gendered language styles may *cause* individuals to favor one narrative over another. Future prospective experiments following up on this research may test whether explicit, implicit, or perceived awareness of the gender of a novelist or screenwriter moderates the relation between linguistic gender congruity and narrative preferences. For instance, when reading novels or stories in everyday life, an author’s gender is likely cued by a combination of the author’s name, the cover’s graphic design, and the writing itself [7]. In order to determine which, if any, gender cues causally influence reader preferences, it will be necessary to experimentally disentangle those variables, as a complement to the present archival analyses. With such a design, we could also obtain information regarding individual differences of raters to examine other potential moderators of the results we have observed, such as audience gender, endorsement of gender stereotypes, or education level.

## Conclusion

The main findings across our two studies suggest that screenwriters’ and novelists’ writing is gender-congruent. Lay audiences and readers alike highly rate gender-congruent films and novels, while professional critics highly rate films with a gender-incongruent writing style. These results may provide insight into the gender imbalance of the film and publishing industries. Audience preferences are one of the major driving forces that influence which films and novels are released and published, respectively. If the masses tend to prefer gender-congruent narratives that perpetuate gendered social roles, then those are the types of narratives that the industry will produce.

The film and publishing industries are notorious for gender inequality. For example, to this day, the name of Alice Guy-Blaché—one of the first ever filmmakers—and her work remain little known [51]. Similarly, female writers, even today, resort to using male names in order to get their books published [52]. Examining how writer gender and gender-linked language relates to narrative receptions strengthens our understanding of the film and publishing industries, paving the road for future experimental research that may further elucidate gender disparities in these industries. Although computational linguistics and other computer science areas are increasingly mining fiction for answers about human psychology, culture, and behavior [53, 54], fictional literature remains an underutilized resource in the behavioral sciences. Our results demonstrate the power of quantitative analyses of fictional text, when paired with complementary archival data on film and novel ratings, to reveal insights about the minds of both authors and audiences.
